# Efficacy of acupuncture in ameliorating anxiety in Parkinson's disease: a systematic review and meta-analysis with trial sequential analysis

**DOI:** 10.3389/fnagi.2024.1462851

**Published:** 2024-11-11

**Authors:** Zhennan Wu, Chang Liu, Vickie Chan, Xiaofeng Wu, Fan Huang, Zining Guo, Wenhao Liu, Liming Lu, Nenggui Xu

**Affiliations:** ^1^South China Research Center for Acupuncture and Moxibustion, Medical College of Acu-Moxi and Rehabilitation, Guangzhou University of Chinese Medicine, Guangzhou, China; ^2^Research Centre of Basic Integrative Medicine, School of Basic Medical Sciences, Guangzhou University of Chinese Medicine, Guangzhou, Guangdong, China; ^3^Department of Rehabilitation Sciences, The Hong Kong Polytechnic University, Kowloon, Hong Kong SAR, China

**Keywords:** Parkinson's disease, anxiety symptoms, acupuncture, meta-analysis, randomized controlled trial

## Abstract

**Background:**

Although numerous studies have explored acupuncture for alleviating Parkinson's disease (PD) symptoms, specific methods focusing on reducing anxiety in these patients are lacking. Preliminary research indicates that acupuncture may improve anxiety in patients with Parkinson's; however, high-quality evidence is lacking. Therefore, we conducted a meta-analysis and trial sequential analysis (TSA) to assess the efficacy of acupuncture in managing anxiety symptoms in PD.

**Methods:**

We systematically searched eight databases for randomized controlled trials (RCTs) evaluating the efficacy of acupuncture for the treatment of anxiety in patients with PD. Primary outcomes were measured using the Hamilton Anxiety Scale (HAMA) and the Self-Rating Anxiety Scale (SAS). Secondary outcomes included the Parkinson's Disease Questionnaire-39 (PDQ-39) and the Unified Parkinson's Disease Rating Scale (UPDRS). Risk of bias was assessed using the Cochrane RoB 2.0 tool, and certainty of evidence was assessed using the GRADE system. The Trial Sequential Analysis (TSA) was used to assess the sufficiency of the evidence.

**Results:**

Our meta-analysis included 14 studies. The Manual acupuncture (MA) + routine drug treatment (RDT) group improved more than the RDT alone group. MA was more effective than sham acupuncture. MA+ traditional Chinese medicine (TCM) was also more effective than TCM. Auricular therapy (AT) was not as effective as control therapy (CT). The Electroacupuncture (EA) + routine drug treatment (RDT) group was not as effective as RDT. PDQ-39 and UPDRS subgroup analysis showed that the acupuncture group had better clinical efficacy than CT. The GRADE assessment rated the overall certainty of evidence for anxiety outcomes as low, PDQ-39 as very low and UPDRS as low. TSA results indicate insufficient evidence; further high-quality RCTs are needed to substantiate these findings.

**Conclusion:**

Our analysis suggests that MA combined with RDT may help ameliorate anxiety in PD patients, although the evidence is weak due to low quality RCTs. EA and AT showed no significant effects, highlighting the need for more rigorous studies with better controls and longer follow-up. The potential of acupuncture for PD-related anxiety should be considered with caution until stronger evidence becomes available.

**Systematic review registration:**

https://www.crd.york.ac.uk/PROSPERO/.

## 1 Introduction

Parkinson's disease (PD) is a common neurodegenerative disorder, typically characterized by motor symptoms such as tremor, bradykinesia, and rigidity (Du et al., 2021). Nevertheless, it has become increasingly evident that non-motor symptoms, particularly anxiety, are exerting an increasingly pivotal influence on the overall impact of the disease (Armstrong and Okun, 2020). It is estimated that approximately one-third of individuals diagnosed with PD also present with anxiety. This has been demonstrated to exacerbate motor symptoms and significantly impair quality of life (Overton and Coizet, 2020; Broen et al., 2016). Concurrently, anxiety has been demonstrated to exacerbate the severity of PD symptoms, affecting patients' mood, cognition and social functioning (Dissanayaka et al., 2010). Neuroimaging research (Li and Xu, 2024) has also explored the connection between anxiety-related brain regions in patients with PD. Individuals with PD often show decreased sizes in the medial prefrontal cortex and anterior cingulate cortex and orbitofrontal cortex, which are regions associated with anxiety. These findings further confirm the association between anatomical changes in the fear circuit and the occurrence of anxiety disorders in patients with PD. However, dysfunction of the fear circuit is not the only mechanism explaining the high prevalence of anxiety in PD. Functional studies indicate that Parkinson's anxiety is also related to the striatum, as well as dopaminergic and noradrenergic pathways, including alterations in the basal ganglia circuits and the cortico-striato-thalamo-cortical circuits. Despite the high prevalence of anxiety in PD, its severity is frequently underestimated, and treatment is often inadequate. It is therefore evident that further research is required in order to develop more effective treatment strategies.

The efficacy of conventional pharmacological interventions in treating anxiety symptoms associated with PD is limited, and they may have adverse effects and complications. A study demonstrated that although antidepressant medications have some therapeutic effects on patients with PD, they have minimal efficacy in treating anxiety disorders (Liu et al., 2013). In addition, the long-term use of these medications may result in adverse effect (Troeung et al., 2013). Moreover, drugs such as levodopa, which are primarily used to improve motor symptoms, have a limited effect on anxiety disorders and may cause motor complications with prolonged administration (Armstrong and Okun, 2020). Consequently, there is a growing interest in complementary and alternative therapies that demonstrate integrative efficacy and minimal side effects.

Acupuncture represents a fundamental aspect of traditional Chinese medicine. At present, acupuncture is primarily employed in the treatment of neurological disorders, musculoskeletal and connective tissue diseases, cancer, and cardiovascular conditions (Lu et al., 2022). The theoretical basis for acupuncture's efficacy in the treatment of anxiety can be traced back to its regulatory effect on the neurotransmitter system. Prior research has demonstrated that acupuncture can alleviate anxiety-related symptoms by regulating the balance of neurotransmitters, including serotonin (Wu et al., 2024), norepinephrine (Zhao et al., 2017), and dopamine (Chen et al., 2023; Wu et al., 2022). Furthermore, acupuncture has been shown to alleviate the anxiety response triggered by stress by modulating the hypothalamic-pituitary-adrenal (HPA) axis (Zheng et al., 2024). In light of these mechanisms, the utilization of acupuncture in the management of anxiety associated with PD is theoretically supported. Recent studies have demonstrated that acupuncture is an efficacious treatment for anxiety disorders in patients with PD (Gu et al., 2023; Chao, 2023; Zhang et al., 2022; Fan et al., 2022).

Although preliminary studies have been conducted on the application of acupuncture, the quality of the existing evidence is inconclusive. Furthermore, there is a paucity of studies on PD-related anxiety. In order to address this research gap, this study employs a systematic review and meta-analysis, combined with a trial sequential analysis, to provide the first comprehensive assessment of the efficacy of acupuncture for the treatment of anxiety in Parkinson's disease. The aim is to establish a more robust evidence base for future clinical treatment.

## 2 Methods

### 2.1 Study registration

This study followed the Preferred Reporting Items for Systematic Reviews and Meta-Analyses (PRISMA) guidelines (Page et al., 2021) and adhered to the PRISMA checklist. It was approved by the International Prospective Register of Systematic Reviews (PROSPERO) on 9 March 2024 under registration number CRD42024518431.

### 2.2 Search strategy

An extensive literature search was conducted using eight databases until 1 March 2024. The databases included four in English (Cochrane Library, PubMed, Embase, Web of Science) and four in Chinese (China Biology Medicine, VIP, Wanfang Data and CNKI). We retrieved publications from all countries and languages, covering various article types. Additionally, we specifically searched for meta-analyses concerning acupuncture treatment for PD. The search utilized key terms such as “Parkinson,” “anxiety,” “acupuncture,” “RCTs,” and “meta-analyses.” A detailed search strategy is provided in Supplementary material 1.

### 2.3 Literature selection

We applied the following set of inclusion criteria during the report selection process (Zhang J. et al., 2024). Inclusion criteria: (1) Type of study: Individuals were diagnosed with PD based on established diagnostic criteria, including the MDS clinical diagnostic criteria for PD (Postuma et al., 2015), the Chinese guidelines for the treatment of PD (second edition; Cheng, 2009), Clinical criteria for diagnosing PD in China (China Medical et al., 2019; Parkinson's et al., 2016), and the accuracy of clinical diagnosis of idiopathic PD (Rajput, 1993). (2) Intervention: acupuncture treatment, including acupuncture alone or combined with Routine drugs treatment (RDT) or control therapy (CT). (3) Comparison: no treatment, sham acupuncture (SA), RDT or CT. (4) The primary outcomes of the study were assessed with the Hamilton Anxiety Scale (HAMA; Hamilton, 1959) and the Self-Rating Anxiety Scale (SAS), which measure anxiety symptoms using specific metrics. The PD Questionnaire (PDQ-39) was used to evaluate the quality of life as a secondary measure. (5) Study design: Randomized controlled trials (RCTs). Language restrictions are English and Chinese. Studies were excluded if they only reported the response rate or lacked data for effect size estimates. Exclusion criteria (Zhang J. et al., 2024): (1) Trials with acupuncture therapy in the control group, (2) Repetitive experiments, (3) Studies involving animal experiments, (4) Studies with incomplete data.

### 2.4 Data extraction

After the search was completed, the retrieved documents were imported into EndNote X9 software. After eliminating duplicates, two reviewers independently examined the titles and abstracts of 41 articles (Wu, Z.N, and Liu, C) to exclude 24 studies that clearly did not meet the inclusion criteria. The complete texts of the remaining studies were reviewed again to determine if the articles satisfied the inclusion criteria. Data extraction was performed by two researchers, capturing details such as the primary author, publication year, sample size, randomization technique, and grouping; participant specifics like gender, age, and illness duration; details on interventions like quantity, acupuncture locations, and EA settings; results data; follow-up results and duration; and any adverse events. In case of disagreements, a third reviewer, Xiaofeng Wu, was brought in to make a final decision.

### 2.5 Quality assessment

The potential for bias in the literature was evaluated using Cochrane RoB2.0 (Sterne et al., 2019). Each RCT was assessed based on six criteria: the randomization procedure, unexpected interventions, missing outcome data, measurement outcomes, reported findings, and overall assessment. A study was classified as low risk if the methodology was appropriate, well-explained, and clear. Conversely, it was deemed high risk if the method was unclear or problematic. Two investigators (Wu, X.F and Huang, F) independently assessed these factors and consulted a third investigator (Vicky Chan) to resolve any differences if necessary.

### 2.6 Statistical analysis

Statistical analysis of the included RCTs was performed using the R “metaphor” package. We assess the effect size by comparing the differences after treatment in each group. Continuous data were presented as mean difference (MD) or standardized mean difference (SMD) with 95% confidence intervals (CI), considering *I*^2^ and *P*-values to assess heterogeneity. When *I*^2^ exceeded 50%, or *P* was < 0.01, indicating significant heterogeneity, a random effects model was utilized; otherwise, a fixed effects model was applied. Subgroup analysis was conducted to identify sources of variability. Sensitivity analysis was performed using leave-one-out analysis to test the robustness of the findings. Each study was systematically excluded one at a time to evaluate its impact on the meta-analysis. In order to enhance the robustness of the results, this study employed the use of trial sequential analysis (TSA). TSA is a statistical method that integrates the traditional interim analysis of RCTs with meta-analysis. In comparison to traditional meta-analysis, TSA is capable of more effectively controlling random errors and type I errors (false positives) through the establishment of the required information size (RIS) and the trial sequential monitoring boundaries (TSMB). TSA was performed using TSA 0.9.5.10 beta (https://www.ctu.dk/tsa/) to calculate the RIS and TSMB. TSA assesses the sufficiency of the extant evidence by calculating the requisite cumulative sample size at a specified α (0.05) and β (0.2) level. Once the accumulated sample size reaches or exceeds the RIS, it indicates that there is sufficient evidence to draw a definitive conclusion. In the event that this is not the case, it is necessary to conduct further high-quality studies in order to verify the results. The use of TSA was selected in order to circumvent the potential for random errors to be introduced by the process of repeated analysis, thereby ensuring that the resulting conclusions are statistically more valid. By establishing a boundary value and the requisite amount of information, TSA offers a more straightforward assessment of the sufficiency and robustness of the evidence, thereby enhancing the credibility of the research conclusions.

### 2.7 Certainty of the evidence

The certainty of evidence was assessed using the Grading of Recommendations Assessment, Development, and Evaluation (GRADE; Guyatt et al., 2008) framework. Evidence levels were categorized as high, moderate, low, or very low based on five components: risk of bias, inconsistency, indirectness, imprecision, and other factors.

## 3 Results

### 3.1 Study selection

**Figure 1:**
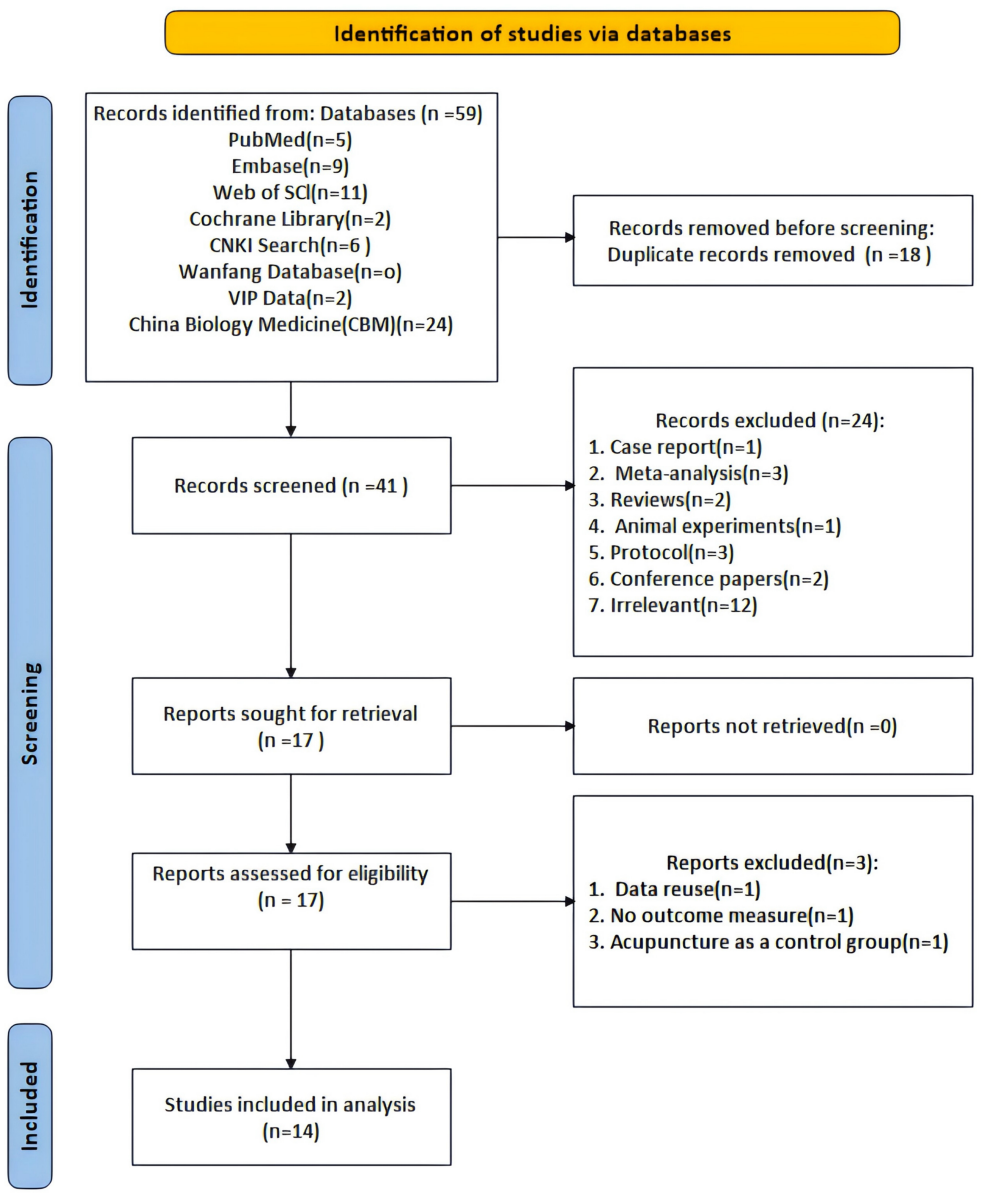
Flow chart of the PRISMA.

Figure 1 shows the PRISMA flowchart for literature screening. We retrieved 59 records from the database. Before filtering, we deleted 18 duplicate records. After screening the titles and abstracts, we selected 41 articles for full-text review after excluding 24 articles based on the inclusion criteria. During the full-text review, three reports were excluded, a total of 45 out of 59 articles were excluded. Finally, 14 articles were included in this meta-analysis and the detailed reasons are shown in the flowchart. For the diagnosis of PD, the MDS clinical diagnostic criteria for PD were used in one study (Fan et al., 2022). For the diagnosis of PD, the Chinese Guidelines for the Treatment of PD (second edition) were used in five studies (Gu et al., 2023; Song, 2021; Bai, 2021; Zhu, 2020; Hu, 2018; Xu, 2017), and the Clinical Criteria for the Diagnosis of PD in China were also used in six studies (Gu et al., 2023; Chao, 2023; Zhang et al., 2022; Li L. et al., 2021; Deng, 2021; Liu, 2020). And for the diagnosis of PD, the accuracy of clinical diagnosis of idiopathic PD was used in two studies (Chen, 2018; Wang et al., 2015).

### 3.2 Study characteristics

#### 3.2.1 Patients

Table 1 summarizes the fundamental details of the included studies. All 14 articles were studies conducted in China between 2015 and 2024. Out of these, two were published in English and the other 12 in Chinese. The total number of eligible cases was 1,175, with 592 in the experimental group and 583 in the control group.

**Table 1:** Characteristics of the included studies.

**References**	**Digotise**	**Randomization**	**Sample Size**	**Average age (mean ±SD)**	**IG**	**CG**	**Intervention**	**Duration (Week)**	**Frequency**	**Acupoints**	**Outcomes**
Fan et al. (2022)	①	SPSS	TG = 32 CG = 32	TG = 61.03 (±9.80) CG = 62.66 (±6.94)	MA	SA	MA	8	Thw	GV 24, GV 29, HT7, SP 6, EX-HN1	HAMA, PDQ39, UPRDS
Deng (2021)	③	RML	TG = 53 CG = 53	TG = 65.12 ± 5.12 CG = 65.12 ± 5.12	MA+TCM	TCM	MA	4	MA:Fiw;TCM:Bid	ST36, ST40, LR3, LI4, GV20, GB20, EX-HN1, GV29, CV24	HAMA, UPRDS
Hu (2018)	②	RD	TG = 55CG = 55	TG = 66.42 ± 4.78 CG = 66.60 ± 4.82	MA+RDT	RDT	MA	24	MA:Qd;TCM:Bid	GB20, BL10, GB12, GV15	SAS, PDQ39
Gu et al. (2023)	③	NR	TG = 52 CG = 51	TG = 51.37 + 7.26 CG = 52.07 ± 7.56	MA+RDT	RDT	MA	12	MA:Fiw;TCM:Qd	GV20, GV29, EX-HN1, PC6, HT7, GB40, PC7	HAMA, UPRDS
Song (2021)	②	RML	TG = 62 CG = 62	TG = 72.64 ± 2.50 CG = 72.53 ± 2.56	MA+RDT	CT	MA	4	MA:Fiw;HBOT:Qd	Dance tremor control area, LI4, LI11, TE5, KI7, ST36, SP6	HAMA
Li L. et al. (2021)	③	RML	TG = 50 CG = 50	TG = 61.56 ± 7.51 CG = 62.49 ± 7.53	EA+RDT	RDT	EA	8	EA:Qd;RDT:Bid	GV20, EX-HN1, LR3, HT7, LI4, SP6, Anmian	SAS
Chen (2018)	④	CE	TG = 30 CG = 29	TG = 67.07 ± 8.75 CG = 63.28 ± 8.78	EA+RDT	RDT	EA	8	EA:Fiw;RRT:Fiw	GV20, GV14	HAMA
Zhang et al. (2022)	③	RML	TG = 43CG = 43	TG = 61.8 ± 4.6 CG = 62.2 ± 4.3	EA+RDT	RDT	EA	4	EA:Fow;RDT:Tid	Dance tremor control area, GV2, EX-HN1, GB20, EX-B2	HAMA, PDQ39
Chao (2023)	③	RML	TG = 50 CG = 50	TG = 70.02 ± 4.27 CG = 69.73 ± 4.92	AT	CT	AT	4	Fod	TF4, AT3, 4 i, CO13, CO10, AT2.3,4 i	SAS, PDQ39
Xu (2017)	②	NR	TG = 51 CG = 52	TG = 71.8 ± 5.3 CG = 73.2 ± 4.9	AT	CT	AT	1.5	Fod	AH6 a, TF4, CO15, CO12	SAS
Liu (2020)	③	RD	TG = 30 CG = 30	TG = 59.31 ± 13 CG = 54.55 ± 1.21	EA+RDT	RDT	EA	4	MA:NR;RDT:Qd	EX-HN1, GV20, GB20, GVl6, LR3	HAMA
Bai (2021)	②	PG	TG = 29 CG = 29	TG = 46–78 CG = 45–79	MA+RDT	RDT	MA	2	MA:Qd:RDT:Qd	GV20, GV29, GB20, EX-HN22, CV6, CV12, ST25, PC7, SP6	HAMA
Wang et al. (2015)	④	RD	TG = 28 CG = 20	TG = 62.1 ± 8.7 CG = 59.1 ± 12.4	EA+RDT	RDT	EA	8	EA:Tow;RDT:Tid	GB20, LI4, GV14, GV16	HAMA
Zhu et al. (2022)	②	RD	TG = 27 CG = 27	TG = 74.2 ± 5.1 CG = 73.6 ± 5.3	MA+TCM	TCM	MA	2	MA:Trw;RDT:Qd	GV20, EX-HN1, GV29, HT7, SP6	SAS

IG, Interventional Group; CG, Controlled Group; NR, Not Reported; RML, Random Number List; CE, Closed Envelope; SPSS, SPSS Randomly Divided; RD, Randomly Divided; PG:Parity grouping; MA, Manual Acupuncture; EA, Electroacupuncture; AT, Auricular Therapy; SA, Sham Acupuncture; RDT, Routine Drug Treatment; TCM, Traditional Chinese Medicine; CT, Conventional Therapy; Qd, Once A Day; Bid, Twice A Day; Tid, Thrice A Day; Thw, Three Times A Week; Fod, Four Times A Day; Tow, Tow times a week; Trw, Three times a week; Fow, Four times a week; Fiw, Five Times A Week; Siw, Six Times A Week; DW, Dilatational Wave; IW, Intermittent Wave; CW, Continuous Wave.

①: MDS clinical diagnostic criteria for Parkinson's disease. ②: Chinese guidelines for the Treatment of Parkinson's disease. ③: Clinical criteria for diagnosing Parkinson's Disease in China. ④: Accuracy of clinical diagnosis of idiopathic Parkinson's disease.

#### 3.2.2 Acupuncture interventions

The studies included various interventions, including manual acupuncture (MA; Gu et al., 2023; Fan et al., 2022; Song, 2021; Bai, 2021; Zhu, 2020; Hu, 2018; Deng, 2021), electroacupuncture (EA; Zhang et al., 2022; Li L. et al., 2021; Liu, 2020; Chen, 2018; Wang et al., 2015), and auricular acupuncture (AT; Chao, 2023; Xu, 2017). Some study groups received AT along with psychological care, while others were treated with EA combined with Routine drugs treatments (RDT). MA was used in combination with RDT in some cases, while other groups received only MA or MA with traditional Chinese medicine (TCM) in several studies. The number of acupoints used ranged from 4 to 10, and most studies followed specific protocols. Treatment duration ranged from 9 days to 6 months, with only one study including follow-up.

#### 3.2.3 Control measures

AT was compared with CT in 2. MA was compared with SA in 1, TCM in 2, RDT in 4. EA was compared with RDT in 5.

### 3.3 Risk of bias

**Figure 2:**
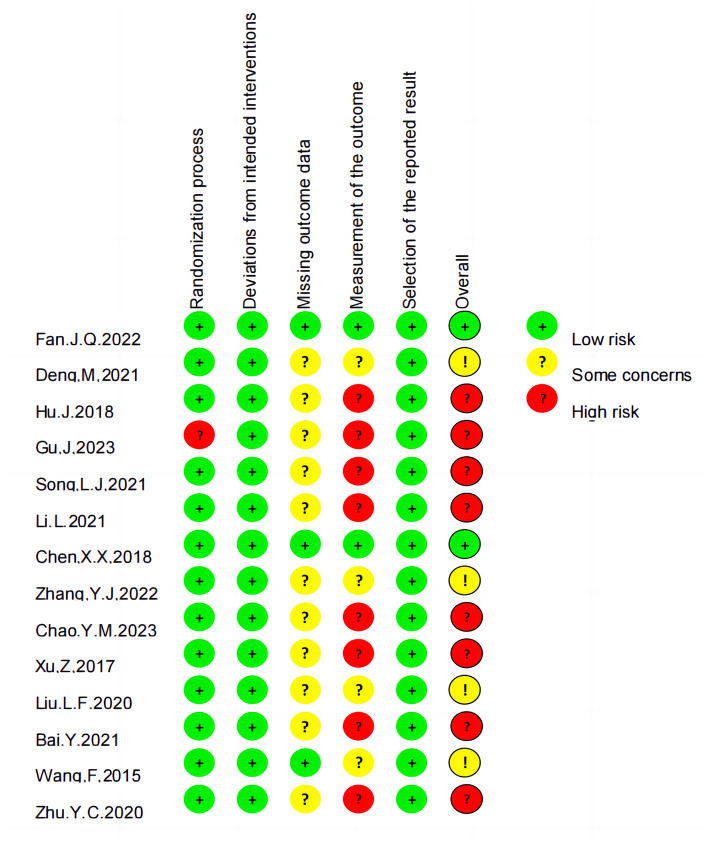
Risk of bias summary.

**Figure 3:**
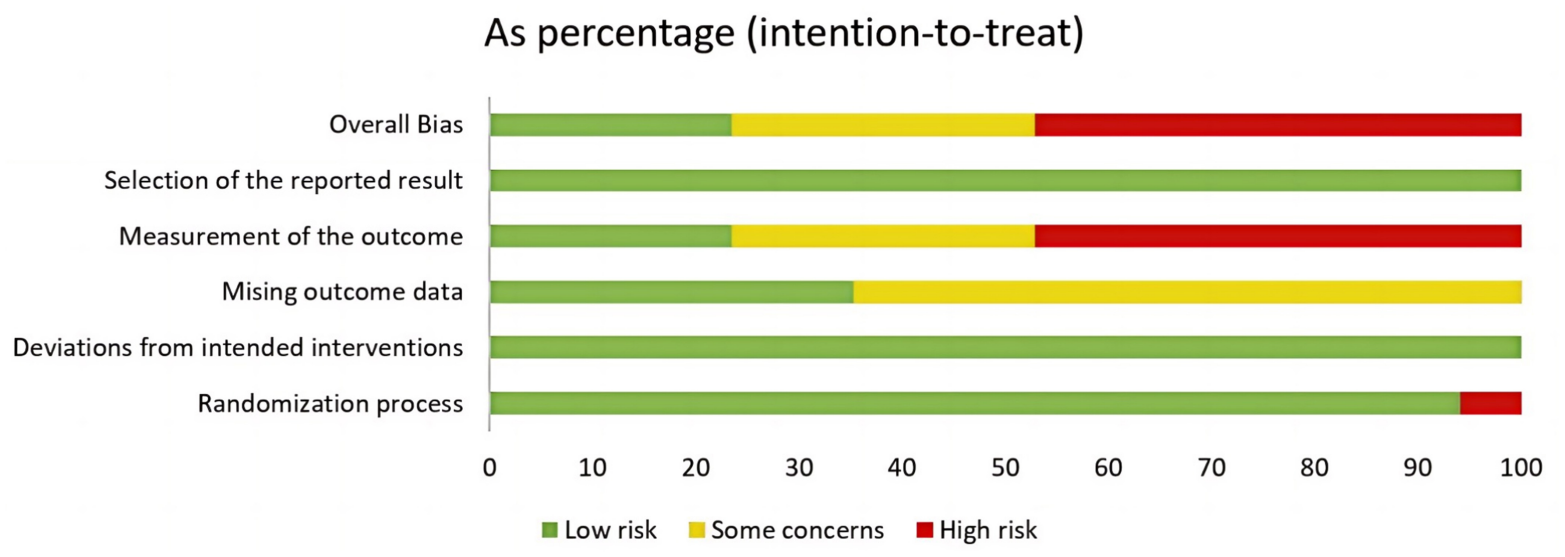
Risk of bias graph.

Among the analyzed RCTs, six were found to have a moderate or low risk of bias, while eight had a high risk of bias, primarily related to randomization or outcome measurement (Figures 2, 3).

### 3.4 Primary outcomes

We assessed the anxiety levels of patients with PD receiving acupuncture treatment using two different scales: the HAMA and the Self-Rating Anxiety Scale. The combined heterogeneity was very high (*I*^2^ = 93%). Sensitivity analysis confirmed the persistent high heterogeneity (Supplementary material 2). We conducted a subgroup analysis to address this, considering that different acupuncture methods might be responsible.

#### 3.4.1 AT vs. CT

This subgroup analysis included two studies demonstrating high heterogeneity, with an *I*^2^ value of 98%. Using a random effects model, the results showed no significant difference in efficacy between the AT and CT groups.

#### 3.4.2 EA + RDT vs. RDT

**Figure 4:**
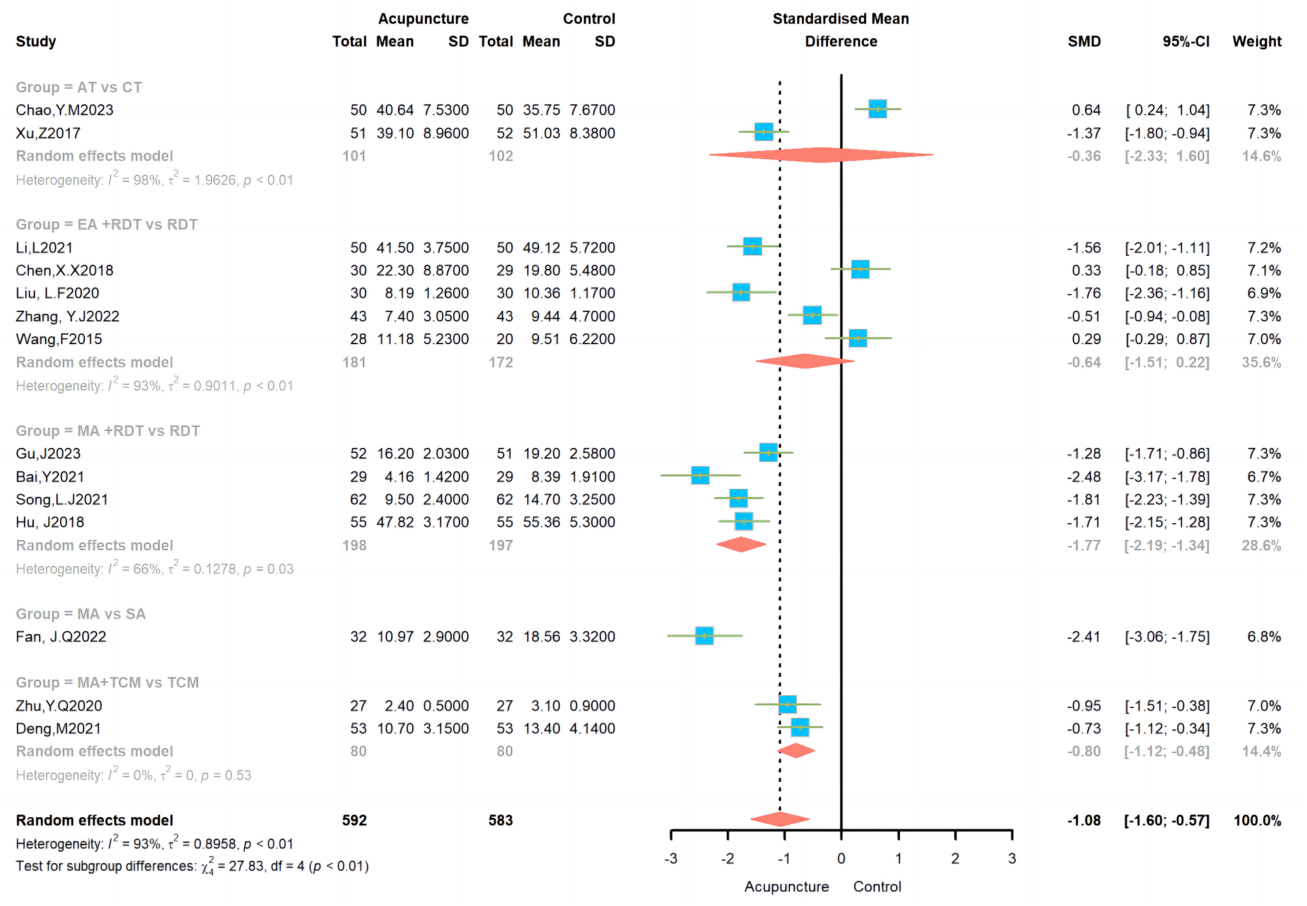
Effects of different acupuncture on anxiety scales.

This subgroup analysis included five studies with high heterogeneity (*I*^2^ = 93 %). Consequently, a random-effects model was applied, and the results indicated no significant difference in efficacy between the EA+RDT and RDT groups (SMD = −0.64, 95% CI = −1.51 – 0.22; Figure 4).

#### 3.4.3 MA + RDT vs. RDT

This subgroup analysis included four studies, with a heterogeneity of *I*^2^ = 66%. A random-effects model was utilized, and the results indicated a significant difference in efficacy between the MA+RDT and RDT groups (SMD = −1.77, 95% CI = −2.19-−1.34; Figure 4).

#### 3.4.4 MA vs. SA

SA was utilized as the control group in only one study within this subgroup. The results suggested a significant difference in efficacy between the MA and SA groups (MD = −2.41, 95% CI = −3.06 – −1.75; Figure 4).

#### 3.4.5 MA + TCM vs. TCM

This subgroup analysis included two studies with no heterogeneity (*I*^2^ = 0%). Consequently, a fixed effects model was utilized, revealing a notable disparity in efficacy between the MA+TCM and TCM groups (SMD = −0.8, 95% CI = −1.12 – −0.48; Figure 4).

### 3.5 Secondary outcomes

#### 3.5.1 PDQ-39

**Figure 5:**
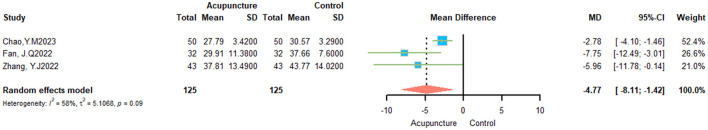
Effects of acupuncture on PDQ-39.

Following the sensitivity analysis (Supplementary material 3), three studies were incorporated into this examination, revealing a diversity of *I*^2^ = 58%. A random effects model showed a significant variation in efficacy between the acupuncture and control groups (SMD = −4.77, 95% CI = −8.11 – −1.42; Figure 5).

#### 3.5.2 UPDRS

**Figure 6:**
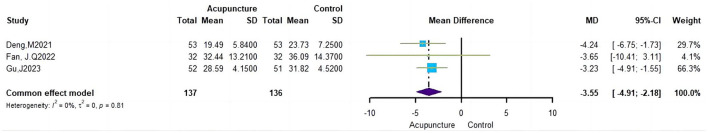
Effects of acupuncture on UPDRS.

After the sensitivity analysis (Supplementary material 4), we excluded one article, resulting in the inclusion of three studies with an *I*^2^ heterogeneity of 0%. Consequently, a fixed effects model was employed, revealing a t significant difference in efficacy between the acupuncture and control groups (MD = −3.55, 95% CI = −4.91 – −2.18; Figure 6).

### 3.6 TSA

**Figure 7:**
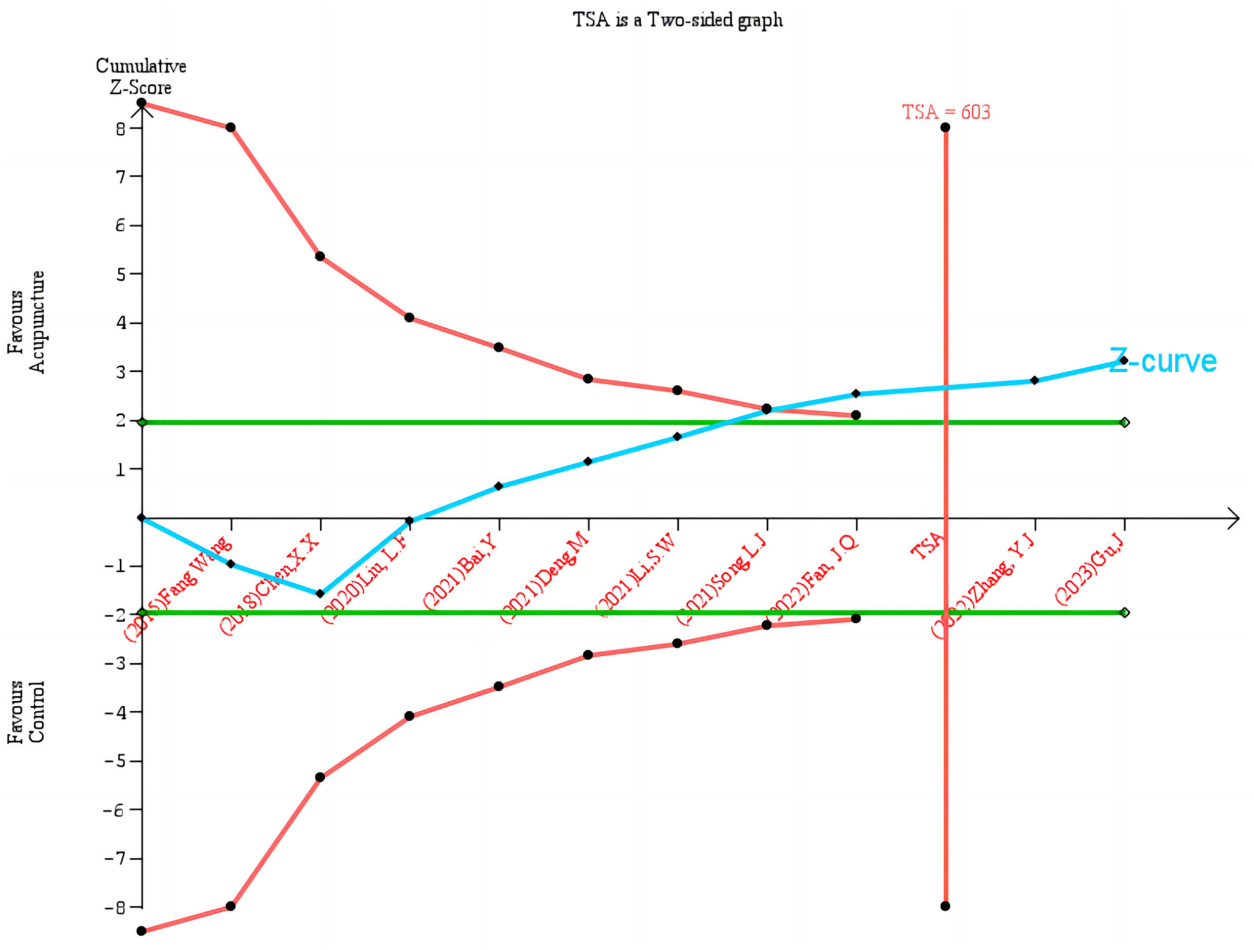
Trial sequential analysis of acupuncture on HAMA.

TSA was used to improve the reliability and robustness of the cumulative evidence. TSA combines interim analyses from classical RCTs with cumulative effect sizes from meta-analyses. It effectively controls for random errors and type I errors (false positives) that can result from repeated analyses, thereby controlling for random errors and type I errors (false positives). TSA ensures statistically valid results by setting a RIS, which is similar to the sample size estimate in individual RCTs. If the cumulative evidence does not reach these thresholds, it indicates that more research is needed to further validate the results. The Z curve (blue line) in the figure demonstrates the dynamic alteration in the cumulative effect size, which gradually approaches the effect boundary (green line) as additional studies are incorporated into the analysis. The red vertical line denotes the quantity of information (603 cases) necessary to achieve the TSA. The figure illustrates that definitive conclusions can be drawn when the cumulative number of studies and sample size reach the RIS (Figure 7).

### 3.7 GRADE

**Figure 8:**
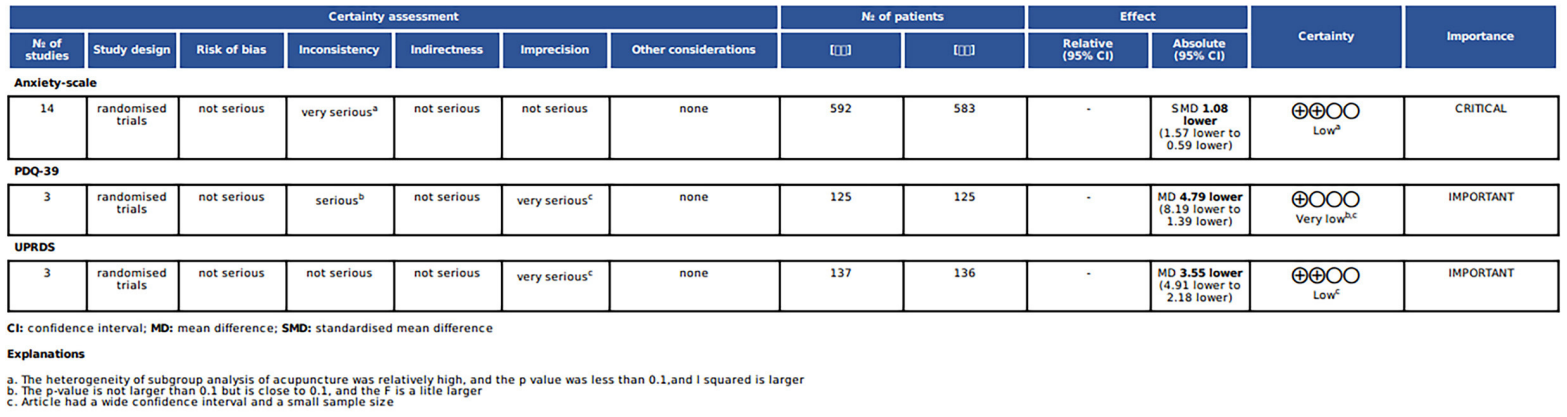
GRADE evidence.

This review assessed the effectiveness of acupuncture for anxiety disorders using the GRADE system and PDQ-39 and UPDRS scores. For anxiety disorders, a review of 14 randomized controlled trials found that acupuncture had a significant effect on reducing anxiety levels, with an SMD of 1.08 (95% CI = 1.57–0.59). Following the GRADE downgrading rules, we found that there were problems with the treatment effect of the acupuncture group in three articles, and there was high heterogeneity. In addition, some of the possible intervals on the anxiety improvement scale were wide, but the sample size was large enough to make the rating serious. Therefore, the evidence rating for this outcome was low. The PDQ-39 and UPDRS were rated very low and low, respectively, and the specific reasons for this are also shown in the graph (Figure 8).

## 4 Discussion

Anxiety in PD may be associated with neurochemical changes in PD, including imbalances in neurotransmitters such as dopamine, 5-hydroxytryptamine (5-HT), and norepinephrine (Khatri et al., 2020). The 5-HT system has seven main receptors and a total of 15 subtypes. If some of the receptors or subtypes in this system are lost or altered, this can have a significant impact on the non-motor symptoms of PD (Muñoz et al., 2020). The activation or blockade of 5-HTR4 in the limbic prefrontal cortex has been demonstrated to improve anxiety-like behavior in rats with PD (Liu et al., 2021). Activation of 5-HT2C receptors in lateral habenular nucleus has been demonstrated to induce or enhance anxiety-like behavior in rats, with this effect being mediated by changes in the levels of monoamine neurotransmitters in relevant brain regions (Han et al., 2016). Furthermore, the ethology of anxiety in PD may be associated with inflammation. Studies have indicated a potential correlation between elevated levels of TNF-α and reduced levels of IL-10 in the amygdala (Ma et al., 2021).

The neurobiological mechanisms of acupuncture in the treatment of anxiety disorders can be described in several ways. Firstly, acupuncture has been demonstrated to relieve anxiety by regulating the neurotransmitter system, particularly the 5-HT system. Anxiety disorders are associated with hyperfunction of the 5-HT system, and acupuncture has been demonstrated to effectively regulate this system, thereby improving anxiety symptoms with fewer side effects (Yuan et al., 2007). There is another viewpoint that the potential mechanism through which acupuncture, 5-HT and anxiety interact may be responsible for the treatment of anxiety in PD (Yutong et al., 2024). Secondly, acupuncture has the capacity to regulate the neuroendocrine system, particularly the HPA axis. Patients with anxiety disorders frequently exhibit dysfunction of the HPA axis, characterized by excessive secretion of adrenocorticotropic hormone and corticosteroids. Acupuncture has been demonstrated to be an effective method for improving anxiety, while also regulating hormone levels, particularly when used in conjunction with pharmacological agents to mitigate adverse effects (Zheng et al., 2024; Zhu et al., 2022). Additionally, acupuncture has been shown to exert a pronounced regulatory influence on the immune system. It is well-established that anxiety disorders are closely associated with immune dysfunction. Acupuncture has the potential to facilitate the restoration of immune function, including improvements in chemotaxis, phagocytosis, lymphocyte proliferation, natural killer cell activity, and superoxide anion levels (Huang et al., 2015; Arranz et al., 2007). Acupuncture has also demonstrated beneficial therapeutic effects in the treatment of PD through mechanisms including oxidative stress-induced apoptosis, mitochondrial dysfunction, autophagy and neurotransmitter regulation (Tamtaji et al., 2019; Jia et al., 2017; Tian et al., 2016; Wang et al., 2013; Zhang et al., 2021). An animal study found that acupuncture can inhibit TNF-α and promote the expression of IL-10 in the amygdala to improve the anxiety symptoms of patients with PD (Ma et al., 2021). The aim of our study was to provide a comprehensive analysis of the efficacy of acupuncture in the treatment of anxiety symptoms in patients with PD.

A sensitivity analysis was initially conducted on the 14 included articles, with I^2^ values ranging from 90 to 94% across studies and an overall *I*^2^ of 93%. This suggests a high degree of heterogeneity across the studies. Meanwhile, the Tau^2^ values ranged from 0.7 to 0.95, and the Tau values ranged from 0.8 to 0.98, indicating a significant variability in effect size. The confidence intervals of the studies did not exhibit exact overlap, further suggesting variability in the results. First, we also considered the possibility that the high heterogeneity could be due to differences in the acupuncture treatment duration (Supplementary material 5). The results showed that whether the acupuncture treatment duration was short-term (< 4 weeks), medium-term (4–8 weeks), or long-term (>8 weeks), the acupuncture group was more effective than the control group in reducing symptoms. However, only the long-term treatment cycle had relatively low heterogeneity, while the short-term and medium-term subgroups had high heterogeneity, which suggests that we need to be cautious when interpreting the results. Therefore, in the articles included in this study, we infer that heterogeneity may be due to the treatment regimens in different studies. A further analysis was therefore conducted on the interventions in question. The results of the study demonstrated that MA combined with RDT exhibited a notable superiority in reducing anxiety levels in comparison to RDT. MA was more effective than sham acupuncture. MA+ TCM was more effective than TCM. However, AT and EA did not demonstrate a statistically significant difference in improving anxiety in PD and displayed no significant efficacy when compared to their respective control treatments. The lack of efficacy of AT and EA merits further discussion. There may be two reasons for the insufficient efficacy of auriculotherapy. First, only two studies were included, which may have affected the reliability of the results of the meta-analysis. In addition, some studies have found that ear stimulation in PD patients mainly acts on gait regulation and motor-related brain activity (Zhang et al., 2023; Marano et al., 2022), so it is also possible that AT is not effective in improving anxiety in PD patients. In terms of treating anxiety in Parkinson's patients, there was no statistically significant difference between EA combined with RDT and RDT alone. The first reason may be that only five articles were included in this paper. The lack of universality may be due to the limited number of studies included in this analysis, and the high heterogeneity could not be reduced through sensitivity analysis. Currently, there is a paucity of research examining the efficacy of EA in alleviating anxiety in patients with PD. This field of study is characterized by a number of limitations and gaps in knowledge. In the five articles included in the review, anxiety was only observed as a secondary indicator. The scales used were HAMA, which was relatively simple, and there was a paucity of scales such as the Beck Anxiety Scale, the Generalized Anxiety Disorder Assessment, and the Global Anxiety Severity and Impairment Scale. Secondly, anxiety is a subjective symptom, and the use of EA, a highly recognized treatment method among the Chinese population, can readily result in a placebo effect during the course of treatment. A mechanistic demonstration of the efficacy of EA was absent in all five included studies. Furthermore, there is currently no standardized treatment plan for the improvement of anxiety in PD patients. The specific operational characteristics of EA, including the selection of acupuncture points, frequency, waveform and current of EA, treatment cycle, and operator proficiency, can all potentially contribute to deviations in experimental results. Additionally, the design and intervention methods employed in each experiment are not uniform, which can significantly impact the reproducibility of experimental outcomes and may also result in high heterogeneity. Therefore, the existing trial data are insufficient to confirm that EA combined with RDT can improve these symptoms in PD patients. A previous meta-analysis of acupuncture for anxiety management also included only one article on EA (Amorim et al., 2018). Conclusions drawn from these limited data may not be widely applicable. Second, most of the current clinical studies on EA focus on improving the motor symptoms of PD (Li et al., 2023), and may be less effective in improving non-motor symptoms. Furthermore, secondary outcomes were examined, including the PDQ-39 and UPDRS. The results demonstrated that acupuncture enhanced the quality of life and alleviated mental symptoms in PD patients, which is consistent with the findings of previous comparable meta-analyses (Fan et al., 2022; Zhu, 2020). However, when interpreting these results, it is essential to consider the sample size and quality of the studies. With relevance to safety, it is notable that only one trial (Fan et al., 2022) reported four adverse reactions to acupuncture, two of which were minor subcutaneous haematomas and two of which were difficulties in removing the needle. This highlights the favorable safety profile of acupuncture treatment.

## 5 Strengths

To the best of our knowledge, this is the first study to examine the impact of acupuncture on anxiety symptoms in individuals with PD. A previous meta-analysis (Li Q. et al., 2022) examined the effect of acupuncture on non-motor symptoms in people with PD, but did not address anxiety specifically. Two meta-analyses (Zhang A. et al., 2024; Zhang Y. et al., 2024) investigated the effect of acupuncture on reducing anxiety symptoms in people with PD. However, these studies were limited to English-language articles, resulting in the inclusion of only two studies. The findings of these studies indicated that acupuncture may not be an effective intervention for improving anxiety symptoms in PD patients. Presently, China is at the vanguard of global clinical acupuncture implementation, with an increasing number of studies examining the impact of acupuncture on anxiety symptoms in PD patients. Consequently, Chinese articles were also incorporated into the meta-analysis. This study combined the data from 14 eligible studies, which assessed patients' anxiety symptoms using the HAMA and SAS scales, and may provide potential evidence for acupuncture as a non-pharmacological treatment for improving anxiety symptoms in PD.

## 6 Limitations of included studies

The included studies have several methodological limitations that may have affected the reliability of the results. These are summarized below: 1. Insufficient randomization and allocation concealment. Some trials lacked a detailed description of their randomization and group concealment procedures, which can lead to selection bias (Gu et al., 2023; Song, 2021; Hu, 2018). If the researcher or subject knows the treatment allocation at the start of the trial, this may affect the balance of the groups and therefore the reliability of the results. 2. Insufficient blinding. Only one article used a double-blind design (Fan et al., 2022), which increases the risk of performance bias and detection bias. The efficacy of acupuncture treatment may be influenced not only by the acupuncture manipulation itself, but also by the expected effects of patients and therapists. Therefore, the lack of a blind design may have overestimated the effectiveness of acupuncture. 3. Small sample sizes and inconsistent research quality. The included studies generally had small sample sizes (Bai, 2021; Zhu, 2020; Wang et al., 2015) and some studies had a risk of bias, such as improper handling of missing data (Wang et al., 2015) and insufficient follow-up (only two articles; Fan et al., 2022; Chen, 2018). This may reduce the statistical power of the results and increase the risk of false positive or false negative results. 4. Heterogeneity of intervention methods. Different studies used different acupuncture methods, such as MA, EA and AT, and there were differences in stimulation intensity, selection of acupuncture points and treatment frequency. This intervention heterogeneity can lead to high heterogeneity of results, which affects our consistent assessment of the efficacy of acupuncture. 5. Lack of standardization and variation in practitioner skills. Inadequate standardization of acupuncture procedures may lead to differences in skill levels between operators. This variation may affect the effectiveness and thus introduce additional variability in results. 6. The possibility of publication bias (Supplementary material 6). As most of the included studies were from China and some were not published in international journals, there is a risk of publication bias. We used the GRADE tool to assess the quality of the evidence and found that the overall quality of the evidence was low, suggesting that more high-quality studies are needed to confirm these findings in the future.

## 7 Implications for clinical practice

**Figure 9:**
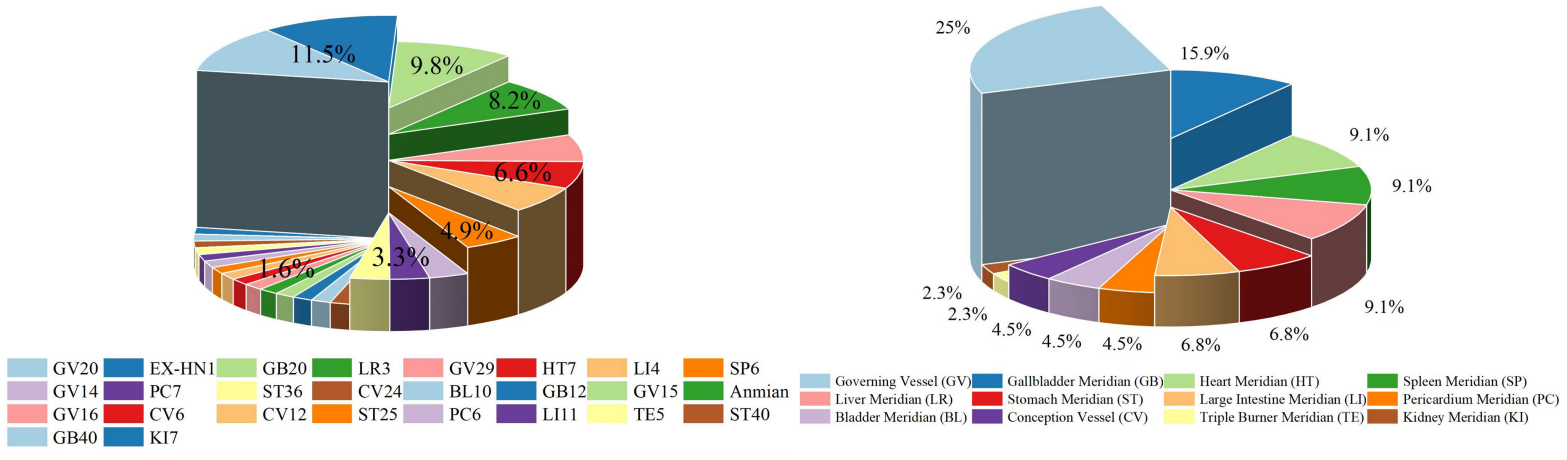
Visualizations of the use of acupuncture points and meridians.

The findings of this study may inform the clinical application of acupuncture for PD anxiety, providing a reference point for the development of a future standardized treatment protocol. Firstly, a summary of the number of acupoints and meridians utilized in the 14 articles was presented (Figure 9). It is worth noting that most articles have utilized the Governing Vessel (GV) and the Gallbladder Meridian (GB), and the majority of articles employed the use of Baihui points (GV20) and Extraordinary head and neck point 1(EX-HN1) and Fengchi (GB20). Among the included studies, GV20, EX-HNT1, and GB20 were chosen because of their wide application in traditional Chinese medicine theory and modern research, particularly for anxiety associated with Parkinson's disease. In TCM theory, the onset of PD is associated with liver and kidney yin deficiency, qi and blood deficiency, and factors such as internal wind, fire, phlegm, and blood stasis. Treatment usually involves tonifying the liver and kidneys, calming the internal wind to stop tremors, and replenishing qi and blood. Anxiety is classified as an “emotional disorder” and is closely related to disorders of the heart, liver, spleen and kidneys. TCM treats anxiety by calming the liver, regulating qi, balancing qi and blood, and balancing yin and yang. GV20 is a key acupoint on the head, closely related to the “heart spirit” and “brain spirit,” and plays a role in regulating mental activity and consciousness. It is highly effective in relieving symptoms such as insomnia, irritability, and heart palpitations caused by anxiety. Modern research has shown that acupuncture at GV meridian can affect mood and cognitive function by regulating the central nervous system (Jingying et al., 2022; Li H. et al., 2022; Xie et al., 2021). EX-HN1 is often used with GV20 to tonify qi, elevate yang, invigorate the brain and calm the mind, which is usually used with GV20 to enhance the sedative and anxiolytic effects by stimulating multiple acupoints. It has shown good results in the treatment of anxiety, memory loss, insomnia, etc, and is used frequently in clinical practice (Li et al., 2021; Liu et al., 2020). GB20 has the functions of pacifying the liver, suppressing yang, dispersing wind and unblocking the meridians. It helps to regulate emotions. Traditional Chinese medicine believes that emotional disorders (such as anxiety and fear) are closely related to liver and gallbladder dysfunction. Therefore, acupuncture at GB20 helps to relieve liver and gallbladder qi and balance the body's qi and blood, thereby regulating emotions. Modern research has found that acupuncture at GB20 can improve blood circulation, increase the supply of oxygen to the brain and regulate the secretion of neurotransmitters (such as dopamine and serotonin), thereby affecting the balance of the central nervous system (Li et al., 2018; Aroxa et al., 2016). The selection of these acupuncture points is based on the theory of traditional Chinese medicine that the liver and gallbladder are associated with emotions, as well as modern research into the role of acupuncture in regulating the central nervous system. In particular, in Parkinson's patients, neurological abnormalities associated with anxiety symptoms are thought to be related to traditional concepts such as liver and gallbladder disharmony and insanity. Acupuncture at these points can regulate the patient's neurological and emotional state, thereby relieving anxiety.

Acupuncture is one of the most important therapies in TCM and is widely used in clinical practice. However, the application and promotion of acupuncture research in the international academic community still faces many challenges. One of the most prominent problems is the lack of standardization in design and implementation. This lack of standardization not only affects the clinical promotion of acupuncture therapy, but also limits its scientific validation and application in the field of medicine. Therefore, regarding the future standardized protocol for acupuncture treatment of PD anxiety, we recommend the following: 1. The intervention methods in acupuncture research need to be standardized. In acupuncture research, factors such as the selection of acupuncture points, the depth of insertion, the manipulation technique, and the retention time can directly affect the therapeutic effect. At present, most clinical acupuncturists mainly use individualized acupoint selection guided by TCM theory, but this can lead to differences in acupuncture methods in different studies, which often makes it difficult to compare results across studies. In order to improve the repeatability and comparability of acupuncture research, acupuncture operations need to be detailed, standardized and unified in the research design. Based on the results of this study, we recommend that in future acupuncture research on the treatment of anxiety-related symptoms of PD, the acupuncture points selected should focus on GV and GB as much as possible; 2. The control design in acupuncture research needs to be standardized as soon as possible. RCTs are the “gold standard” for clinical verification of acupuncture therapy, but in acupuncture research, selecting an appropriate control group is a major challenge. Common control methods include the use of placebo needles, routine treatment controls or blank controls. Different control methods can lead to very different interpretations of the research results. Although some studies have found that placebo needles may have a placebo effect (Wang, 2024), they are currently the best control method because they can better control psychological effects and enhance the internal validity of the experimental results. Therefore, in the design of acupuncture studies, it is recommended to select a placebo control group to ensure the reliability of the results; 3. The evaluation criteria for the efficacy of acupuncture usually rely on subjective indicators such as improvement of symptoms and functional assessment, which are highly subjective. To improve the objectivity of the research results, it is recommended to combine objective biomarkers and clinical indicators, such as biochemical test data of serum inflammatory factors, neurotransmitter levels (Fan et al., 2022); 4. A multi-center, large-sample study design can further improve the external validity and generalizability of the research results. Standardizations of data analysis and reporting is also important. In addition to following conventional statistical methods, special attention should be paid to the application of advanced statistical methods such as multiple test correction and effect size analysis in the statistical analysis of acupuncture studies (Lu et al., 2024). The use of data sharing platforms should be encouraged to promote data transparency and academic exchange; 5. Future clinical research designs should incorporate more rigorous follow-up feedback to enable the evaluation of the medium- and long-term efficacy of acupuncture in a more scientific and objective manner, particularly in the context of chronic neurological conditions such as PD.

In conclusion, acupuncture shows promise for alleviating anxiety in PD by targeting key acupoints such as GV20, EX-HN1, and GB20, which regulate emotional and neurological function. However, challenges in standardizing research design, control groups and outcome measures limit their clinical validation. Future studies should focus on developing standardized protocols, incorporating objective biomarkers, using placebo-controlled RCTs, and conducting multicentre, large-sample trials. Rigorous follow-up and advanced statistical methods will further increase the reliability and applicability of the results and promote the global acceptance of acupuncture for PD-related anxiety.

## 8 Conclusion

Our analysis suggests that MA holds promise for ameliorating anxiety in patients with PD, especially when combined with RDT. However, the evidence supporting these findings is limited by the overall low quality of the included randomized controlled trials RCTs, which reduces confidence in the observed benefits. In contrast, EA and AT did not show significant efficacy in reducing anxiety symptoms. This suggests that future studies need to investigate these methods further, with an emphasis on high-quality, rigorously designed RCTs that include robust controls, including sham acupuncture, and longer follow-up periods to assess long-term outcomes. Until more conclusive evidence is available, the therapeutic potential of acupuncture for PD-related anxiety should be interpreted with caution.

## Data Availability

The original contributions presented in the study are included in the article/Supplementary material, further inquiries can be directed to the corresponding authors.

## References

[B1] Amorim D. Amado J. Brito I. Fiuza S. M. Amorim N. Costeira C. . (2018). Acupuncture and electroacupuncture for anxiety disorders: a systematic review of the clinical research. Complement. Ther. Clin. Pract. 31, 31–37. 10.1016/j.ctcp.01008 29705474

[B2] Armstrong M. J. Okun M. S. (2020). Diagnosis and treatment of Parkinson disease: a review. J. Am. Med. Assoc. 323, 548–560. 10.1001/jama.2019.22360 32044947

[B3] Aroxa F. H. Gondim I. T. Santos E. L. Coriolano M. D. Asano A. G. Asano N. M. . (2016). Acupuncture as adjuvant therapy for sleep disorders in Parkinson's disease. J. Acupunct. Meridian Stud. 10, 33–38. 10.1016/j.jams.12007 28254099

[B4] Arranz L. Guayerbas N. Siboni L. De la Fuente M. (2007). Effect of acupuncture treatment on the immune function impairment found in anxious women. Am. J. Chin. Med. 35, 35–51. 10.1142/S0192415X07004606 17265549

[B5] Bai Y. (2021). Acupuncture treatment for 56 cases of Parkinson's disease with insomnia: a clinical observation. Mov. Disord. 28, 506–507.

[B6] Broen M. P. Narayen N. E. Kuijf M. L. Dissanayaka N. N. Leentjens A. F. (2016). Prevalence of anxiety in Parkinson's disease: a systematic review and meta-analysis. Mov. Disord. 31, 1125–1133. 10.1002/mds.26643 27125963

[B7] Chao Y. M. (2023). Application effectiveness of auricular acupressure combined with mind mapping and cognitive behavioral therapy on Parkinson's syndrome and its impact on negative emotions in patients. Zhonghua Yangsheng Baojian 41, 33–36.

[B8] Chen L. Liu Z. Zhao Z. Du D. Pan W. Wei X. . (2023). Dopamine receptor 1 on CaMKII-positive neurons within claustrum mediates adolescent cocaine exposure-induced anxiety-like behaviors and electro-acupuncture therapy. Theranostics 13, 3149–3164. 10.7150/thno.83079 37351159 PMC10283049

[B9] Chen X. X. (2018). Acupuncture at Baihui and Da Zhui Points Combined With Rehabilitation Robot Training for Non-motor Symptoms of Early Parkinson's Disease. Zhejiang: Zhe Jiang Chinese Medical University.

[B10] Cheng S. D. (2009). Chinese guidelines for the treatment of Parkinson′s disease (second edition). Chin. J. Neurol. 42:4.

[B11] China Medical A. Chinese Medical Journals Publishing House Co L. Chinese Society of General P. Editorial Committee of the Chinese Journal of General Practitioners of the Chinese Medical A. Diseases Egotpogfpd Treatment of N. (2019). Primary diagnosis and treatment guidelines for Parkinson's disease. Chin. J. Gen. Pract. 19, 5–17.

[B12] Deng M. (2021). Effects of Dingchan Anshen formula combined with acupuncture on mild cognitive function and neurotransmitters levels in patients with Parkinson disease. Chin. For. Med. Res. 19, 128–131.

[B13] Dissanayaka N. N. Sellbach A. Matheson S. O'Sullivan J. D. Silburn P. A. Byrne G. J. . (2010). Anxiety disorders in Parkinson's disease: prevalence and risk factors. Mov. Disord. 25, 838–845. 10.1002/mds.22833 20461800

[B14] Du T. Wang L. Liu W. Zhu G. Chen Y. Zhang J. . (2021). Biomarkers and the role of alpha-synuclein in Parkinson's disease. Front. Aging Neurosci. 13:645996. 10.3389/fnagi.2021.645996 33833675 PMC8021696

[B15] Fan J. Q. Lu W. J. Tan W. Q. Liu X. Wang Y. T. Wang N. B. . (2022). Effectiveness of acupuncture for anxiety among patients with Parkinson disease: a randomized clinical trial. J. Am. Med. Assoc. Netw. Open 5:E2232133. 10.1001/jamanetworkopen.2022.32133 36129711 PMC9494193

[B16] Gu J. Shen L. R. Wang Q. D. (2023). Clinical study on acupuncture treatment of Parkinson's disease with obsessive state. Chin. Prim. Health Care 37, 89–91.38840478

[B17] Guyatt G. H. Oxman A. D. Vist G. E. Kunz R. Falck-Ytter Y. Alonso-Coello P. . (2008). GRADE: an emerging consensus on rating quality of evidence and strength of recommendations. Br. Med. J. 336, 924–926. 10.1136/bmj.39489.470347.AD 18436948 PMC2335261

[B18] Hamilton M. (1959). The assessment of anxiety states by rating. Br. J. Med. Psychol. 32, 50–55. 10.1111/j.2044-8341.1959.tb00467.x 13638508

[B19] Han L. N. Wang C. L. Zhang L. (2016). Requlatory effects of 5-HT2c receptors in lateral habenular nucleus on anxiety behaviors in rat models of Parkinson's disease and their mechanisms. J. Jilin Univ. 42, 473–480.

[B20] Hu J. (2018). Curative effect of traditional Chinese drugs for Bushen Tongqiao combined with acupuncture in the treatment of Parkinson syndrome of phlegm and blood stasis syndrome and its influence on oxidative stress index. Mod. J. Integr. Trad. Chin. West. Med. 27, 946–949.

[B21] Huang W. Q. Zhou Q. Z. Liu X. G. Wei D. N. He W. Zhang X. D. . (2015). Effects of acupuncture intervention on levels of T lymphocyte subsets in plasma and thymus in stress-induced anxiety rats. Zhen Ci Yan Jiu. 40, 265–269. 26502538

[B22] Jia Y. J. Deng J. H. Zhang W. Z. Sun Z. L. Yang J. Yu Y. . (2017). The role of group II metabotropic glutamate receptors in the striatum in electroacupuncture treatment of parkinsonian rats. CNS Neurosci. Ther. 23, 23–32. 10.1111/cns.12587 27412260 PMC6492692

[B23] Jingying Z. Zhiyi L. Guoshan Z. (2022). Clinical acupoint selection rule of acupuncture and moxibustion in treating Parkinson's disease. Chin. Med. Mod. Dist. Educ. China 20, 49–52.

[B24] Khatri D. K. Choudhary M. Sood A. Singh S. B. (2020). Anxiety: an ignored aspect of Parkinson's disease lacking attention. Biomed. Pharmacother. 131:110776. 10.1016/j.biopha.2020.110776 33152935

[B25] Li F. Wang Y. Jiang T. X. Zhu M. J. Ji J. J. Wu W. W. . (2021). Acupuncture and moxibustion for vascular dementia and its effect on serum VEGF and AChE. Zhongguo Zhen Jiu 41, 851–854. 34369693 10.13703/j.0255-2930.20200816-0001

[B26] Li H. Lai L. Li X. Wang R. Fang X. Xu N. . (2022). Electroacupuncture ameliorates cognitive impairment by regulating gamma-amino butyric acidergic interneurons in the hippocampus of 5 familial Alzheimer's disease mice. Neuromodulation (2024) 27, 730–741. 10.1016/j.neurom.11014 36604241

[B27] Li K. Xu S. Wang R. Zou X. Liu H. Fan C. . (2023). Electroacupuncture for motor dysfunction and constipation in patients with Parkinson's disease: a randomised controlled multi-centre trial. EClinicalMedicine 56:101814. 10.1016/j.eclinm.2022.101814 36691434 PMC9860357

[B28] Li L. Tian Z. W. Zhang X. (2021). Clinical study on electroacupuncture combined with madopar for sleep disorders in Parkinson disease. J. N. Chin. Med. 53, 113–116.

[B29] Li Q. Wu C. Wang X. Li Z. Hao X. Zhao L. . (2022). Effect of acupuncture for non-motor symptoms in patients with Parkinson's disease: a systematic review and meta-analysis. Front. Aging Neurosci. 14:995850. 10.3389/fnagi.2022.995850 36275001 PMC9582755

[B30] Li Y. X. Xu B. C. (2024). Research progress in neuroimaging for Parkinson's disease with anxiety disorder. Chin. J. Pract. Nerv. Dis. 27, 920–924.

[B31] Li Z. Chen J. Cheng J. Huang S. Hu Y. Wu Y. . (2018). Acupuncture modulates the cerebello-thalamo-cortical circuit and cognitive brain regions in patients of Parkinson's disease with tremor. Front. Aging Neurosci. 10:206. 10.3389/fnagi.2018.00206 30034336 PMC6043808

[B32] Liu E. J. Zhang W. L. Wang J. B. Zhao F. G. Bai Y. P. (2020). Acupuncture combined with cranial electrotherapy stimulation on generalized anxiety disorder: a randomized controlled trial. Zhongguo Zhen Jiu 40, 1187–1190. 33788486 10.13703/j.0255-2930.20190917-k0004

[B33] Liu J. Dong J. Wang L. Su Y. Yan P. Sun S. . (2013). Comparative efficacy and acceptability of antidepressants in Parkinson's disease: a network meta-analysis. PLoS ONE 8:e76651. 10.1371/journal.pone.0076651 24098546 PMC3788746

[B34] Liu L. F. (2020). The clinical efficacy of duloxetine combined with acupuncture in treating anxiety in Parkinson's disease. Nei Mongol J. Trad. Chin. Med. 39, 137–138.

[B35] Liu Y. W. Ma H. Y. Gao F. Wang Z. L. Hou Y. L. Cui C. K. . (2021). Improvement effect of activation and blocking of 5-HTR4 inPrL region on anxiety behaviors in Parkinson 's disease rats. J. Jilin Univ. 47, 25–34.

[B36] Lu L. Chen C. Chen Y. Dong Y. Chen R. Wei X. . (2024). Effect of acupuncture for methadone reduction: a randomized clinical trial. Ann. Intern. Med. 177, 1039–1047. 10.7326/M23-2721 38976882

[B37] Lu L. Zhang Y. Ge S. Wen H. Tang X. Zeng J. C. . (2022). Evidence mapping and overview of systematic reviews of the effects of acupuncture therapies. Br. Med. J. Open 12:e056803. 10.1136/bmjopen-2021-056803 35667716 PMC9171228

[B38] Ma Z. W. Liang H. Zhao B. Cai S. J. Guo J. Yu D. . (2021). Effect of acupoint piercing on expression of TNF-α and lL-10 in amygdala of anxious rats with Parkinson's disease. Shaanxi J. Trad. Chin. Med. 42, 1171–1174.

[B39] Marano M. Anzini G. Musumeci G. Magliozzi A. Pozzilli V. Capone F. . (2022). Transcutaneous auricular vagus stimulation improves gait and reaction time in Parkinson's disease. Mov. Disord. 37, 2163–2164. 10.1002/mds.29166 35861362 PMC9796229

[B40] Muñoz A. Lopez-Lopez A. Labandeira C. M. Labandeira-Garcia J. L. (2020). Interactions between the serotonergic and other neurotransmitter systems in the Basal Ganglia: role in Parkinson's disease and adverse effects of L-DOPA. Front. Neuroanat. 14:26. 10.3389/fnana.2020.00026 32581728 PMC7289026

[B41] Overton P. G. Coizet V. (2020). The neuropathological basis of anxiety in Parkinson's disease. Med. Hypotheses 144:110048. 10.1016/j.mehy.2020.110048 32758886

[B42] Page M. J. McKenzie J. E. Bossuyt P. M. Boutron I. Hoffmann T. C. Mulrow C. D. . (2021). The PRISMA 2020 statement: an updated guideline for reporting systematic reviews. Br. Med. J. 372:n71. 10.1136/bmj.n71 33782057 PMC8005924

[B43] Parkinson's D. Movement Disorders Group of the Neurology Section of the Chinese Medical A. Specialized Committee on Parkinson's D. Movement Disorders N. B. C. P. A. (2016). Clinical diagnostic criteria for Parkinson disease in China. Chin. J. Neurol. 49, 268–271.

[B44] Postuma R. B. Berg D. Stern M. Poewe W. Olanow C. W. Oertel W. . (2015). MDS clinical diagnostic criteria for Parkinson's disease. Mov. Disord. 30, 1591–1601. 10.1002/mds.26424 26474316

[B45] Rajput D. R. (1993). Accuracy of clinical diagnosis of idiopathic Parkinson's disease. J. Neurol. Neurosurg. Psychiatry 56, 938–939. 10.1136/jnnp.56.8.938 8350122 PMC1015162

[B46] Song L. J. (2021). Effects of acupuncture and moxibustion combined with hyperbaric oxygen and rehabilitation exercise on non-motor symptoms and mental state of patients with Parkinson's disease. Reflexol. Rehabil. Med. 2, 13–15.

[B47] Sterne J. A. C. Savovic J. Page M. J. Elbers R. G. Blencowe N. S. Boutron I. . (2019). RoB 2: a revised tool for assessing risk of bias in randomised trials. Br. Med. J. 366:l4898. 10.1136/bmj.l4898 31462531

[B48] Tamtaji O. R. Naderi Taheri M. Notghi F. Alipoor R. Bouzari R. Asemi Z. . (2019). The effects of acupuncture and electroacupuncture on Parkinson's disease: current status and future perspectives for molecular mechanisms. J. Cell Biochem. 120, 12156–12166. 10.1002/jcb.28654 30938859

[B49] Tian T. Sun Y. Wu H. Pei J. Zhang J. Zhang Y. . (2016). Acupuncture promotes mTOR-independent autophagic clearance of aggregation-prone proteins in mouse brain. Sci. Rep. 6:19714. 10.1038/srep19714 26792101 PMC4726430

[B50] Troeung L. Egan S. J. Gasson N. (2013). A meta-analysis of randomised placebo-controlled treatment trials for depression and anxiety in Parkinson's disease. PLoS ONE 8:e79510. 10.1371/journal.pone.0079510 24236141 PMC3827386

[B51] Wang F. Sun L. Zhang X. Z. Jia J. Liu Z. Huang X. Y. . (2015). Effect and potential mechanism of electroacupuncture add-on treatment in patients with Parkinson's disease. Evid. Based Complement. Alternat. Med. 2015:692795. 10.1155/2015/692795 26351515 PMC4550783

[B52] Wang H. Liang X. Wang X. Luo D. Jia J. Wang X. . (2013). Electro-acupuncture stimulation improves spontaneous locomotor hyperactivity in MPTP intoxicated mice. PLoS ONE 8:e64403. 10.1371/journal.pone.0064403 23737982 PMC3667805

[B53] Wang Y. T. (2024). Analysis of placebo effect of acupuncture. Acupunct. Res. 49, 875–879. 10.13702/j.1000-0607.20230390 39318318

[B54] Wu M. Chen Y. Shen Z. Zhu Y. Xiao S. Zhu X. . (2022). Electroacupuncture alleviates anxiety-like behaviors induced by chronic neuropathic pain via regulating different dopamine receptors of the basolateral amygdala. Mol. Neurobiol. 59, 5299–5311. 10.1007/s12035-022-02911-6 35696012 PMC9395447

[B55] Wu Z. Shen Z. Xu Y. Chen S. Xiao S. Ye J. . (2024). A neural circuit associated with anxiety-like behaviors induced by chronic inflammatory pain and the anxiolytic effects of electroacupuncture. CNS Neurosci. Ther. 30:e14520. 10.1111/cns.14520 38018559 PMC11017463

[B56] Xie L. Liu Y. Zhang N. Li C. Sandhu A. F. Williams G. . (2021). Electroacupuncture improves M2 microglia polarization and glia anti-inflammation of hippocampus in Alzheimer's disease. Front. Neurosci. 15:689629. 10.3389/fnins.2021.689629 34646113 PMC8502881

[B57] Xu Z. (2017). The effects of auricular seed embedding combined with psychological nursing on anxiety and depression in patients with Parkinson's disease. Chin. Manipul. Rehabil. Med. 8, 73–74.

[B58] Yuan Q. Li J. N. Liu B. Wu Z. F. Jin R. (2007). Effect of Jin-3-needling therapy on plasma corticosteroid, adrenocorticotrophic hormone and platelet 5-HT levels in patients with generalized anxiety disorder. Chin. J. Integr. Med. 13, 264–268. 10.1007/s11655-007-0264-9 18180890

[B59] Yutong Z. Lin B. Xiaoning L. (2024). Research progress of acupuncture in the treatment of Parkinson's disease related pathogenesis. J. Liaoning Univ. Trad. Chin. Med. 26, 101–106.

[B60] Zhang A. Song Z. Di A. Zhou Z. Zheng L. Zhuang L. . (2024). Acupuncture for the treatment of neuropsychiatric symptoms in Parkinson's disease: a systematic review and meta-analysis. Complement. Ther. Med. 80:103020. 10.1016/j.ctim.2024.103020 38185400

[B61] Zhang H. Cao X. Y. Wang L. N. Tong Q. Sun H. M. Gan C. T. . (2023). Transcutaneous auricular vagus nerve stimulation improves gait and cortical activity in Parkinson's disease: a pilot randomized study. CNS Neurosci. Ther. 29, 3889–3900. 10.1111/cns.14309 37311693 PMC10651956

[B62] Zhang J. Yang X. Zhang X. Lu D. Guo R. (2021). Electro-acupuncture protects diabetic nephropathy-induced inflammation through suppression of NLRP3 inflammasome in renal macrophage isolation. Endocr. Metab. Immune Disord. Drug Targets 21, 2075–2083. 10.2174/1871530321666210118161721 33461476

[B63] Zhang J. Zhou X. Jiang H. Zhu W. Chi H. Jiang L. . (2024). Acupuncture for insomnia symptoms in hypertensive patients: a systematic review and meta-analysis. Front. Neurol. 15:1329132. 10.3389/fneur.2024.1329132 38440112 PMC10910107

[B64] Zhang Y. Liu S. Xu K. Zhou Y. Shen Y. Liu Z. . (2024). Non-pharmacological therapies for treating non-motor symptoms in patients with Parkinson's disease: a systematic review and meta-analysis. Front. Aging Neurosci. 16:1363115. 10.3389/fnagi.2024.1363115 38737585 PMC11082280

[B65] Zhang Y. J. Qi F. Y. Jin Y. (2022). The short-term effects of scalp acupuncture combined with transcutaneous electrical acupoint stimulation on non-motor symptoms and quality of life in early Parkinson's disease patients. Mod. J. Integr. Trad. Chin. West. Med. 31, 3322–3326.

[B66] Zhao Z. Kim S. C. Liu H. Zhang J. Wang Y. Cho I. J. . (2017). Manual acupuncture at PC6 ameliorates acute restraint stress-induced anxiety in rats by normalizing amygdaloid noradrenergic response. Evid. Based Complement. Alternat. Med. 2017:4351723. 10.1155/2017/4351723 28900460 PMC5576413

[B67] Zheng J. Wang Y. Zhang C. Zhang A. Zhou Y. Xu Y. . (2024). Electroacupuncture negatively regulates the Nesfatin-1/ERK/CREB pathway to alleviate HPA axis hyperactivity and anxiety-like behaviors caused by surgical trauma. Chin. Med. 19:108. 10.1186/s13020-024-00974-2 39153974 PMC11330601

[B68] Zhu J. Wang C. Wang Y. Guo C. Lu P. Mou F. . (2022). Electroacupuncture alleviates anxiety and modulates amygdala CRH/CRHR1 signaling in single prolonged stress mice. Acupunct. Med. 40, 369–378. 10.1177/09645284211056352 35044840

[B69] Zhu Y. C. (2020). The clinical observation of modified Gui Pi Tang combined with acupuncture in treating insomnia of Parkinson's disease with heart and spleen deficiency type. Guangming J. Chin. Med. 35, 3253–3255.

