# Supplementary material for: Efficacy of acupuncture in ameliorating anxiety in Parkinson's disease: a systematic review and meta-analysis with trial sequential analysis
Source: Front Aging Neurosci. 2024 Nov 11;16:1462851. doi: 10.3389/fnagi.2024.1462851 (PMC11586373; doi:10.3389/fnagi.2024.1462851)
Supplement: Supplementary file 1 [file Data_Sheet_1.docx]

**Table 1:** PubMed Search

| **NO** | **Search Details** | **Results** |
| --- | --- | --- |
| #1 | "Acupuncture"[Mesh] OR "Acupuncture Therapy"[Mesh] OR "Acupuncture, Ear"[Mesh] OR "Acupuncture Points"[Mesh] OR "Acupuncture Analgesia"[Mesh] | 30,687 |
| #2 | ((((((((((Pharmacopuncture) OR (Acupuncture Treatment)) OR (Acupuncture Treatments)) OR (Treatment, Acupuncture)) OR (Pharmacoacupuncture Treatment)) OR (Pharmacoacupuncture Therapy)) OR (Acupotomies)) OR (Acupuncture Point)) OR (Acupoint)) OR (Acupuncture Anesthesia)) OR (Anesthesia, Acupuncture) | 44,808 |
| #3 | #1 OR #2 | 44,808 |
| #4 | "Parkinson Disease"[Mesh] | 84,473 |
| #5 | ((((((((((Idiopathic Parkinson's Disease) OR (Lewy Body Parkinson's Disease)) OR (Parkinson's Disease, Idiopathic)) OR (Parkinson's Disease, Lewy Body)) OR (Parkinson Disease, Idiopathic)) OR (Parkinson's Disease)) OR (Idiopathic Parkinson Disease)) OR (Lewy Body Parkinson Disease)) OR (Primary Parkinsonism)) OR (Parkinsonism, Primary)) OR (Paralysis Agitans) | 147,502 |
| #6 | #4 OR #5 | 147,502 |
| #7 | "Anxiety"[Mesh] | 115,697 |
| #8 | ((((((Angst[Title) OR (Social Anxiety)) OR (Anxieties, Social)) OR (Social Anxieties)) OR (Hypervigilance)) OR (Nervousness)) OR (Anxiousness) | 334,368 |
| #9 | #7 OR #8 | 334,368 |
| #10 | #6 AND #9 | 3,568 |
| #11 | "Randomized Controlled Trial" [Publication Type] | 609,619 |
| #12 | ((Clinical Trials, Randomized) OR (Trials, Randomized Clinical)) OR (Controlled Clinical Trials, Randomized) | 772,202 |
| #13 | #11 OR #12 | 772,202 |
| #14 | #3 AND #10 AND #13 | 5 |

**Table 2:** EMbase Search

| **NO** | **Search Details** | **Results** |
| --- | --- | --- |
| #1 | 'parkinson disease'/exp | 201647 |
| #2 | 'idiopathic parkinss disease':ab,ti OR 'lewy body parkinsons disease':ab,ti OR 'parkinsons disease, idiopathic':ab,ti OR 'parkinsons disease, lewy body':ab,ti OR 'parkinson disease, idiopathic':ab,ti OR 'parkinsons disease':ab,ti OR 'idiopathic parkinson disease':ab,ti OR 'lewy body parkinson disease':ab,ti OR 'primary parkinsonism':ab,ti OR 'parkinsonism, primary':ab,ti OR 'paralysis agitans':ab,ti | 1947 |
| #3 | #1 OR #2 | 201945 |
| #4 | anxiety | 533397 |
| #5 | 'angst':ab,ti OR 'social anxiety':ab,ti OR 'anxiety, social':ab,ti OR 'anxieties, social':ab,ti OR 'social anxieties':ab,ti OR 'hypervigilance':ab,ti OR 'nervousness':ab,ti OR 'anxiousness':ab,ti | 16619 |
| #6 | #4 OR #5 | 536719 |
| #7 | 'acupuncture':ab,ti OR 'acupuncture therapy':ab,ti OR 'acupuncture, ear':ab,ti OR 'acupuncture points':ab,ti OR 'acupuncture analgesia':ab,ti | 39385 |
| #8 | 'pharmacopuncture':ab,ti OR 'acupuncture treatment':ab,ti OR 'acupuncture treatments':ab,ti OR 'treatment, acupuncture':ab,ti OR 'therapy, acupuncture':ab,ti OR 'pharmacoacupuncture treatment':ab,ti OR 'treatment, pharmacoacupuncture':ab,ti OR 'pharmacoacupuncture therapy':ab,ti OR 'therapy, pharmacoacupuncture':ab,ti OR 'acupotomy':ab,ti OR 'acupotomies':ab,ti OR 'acupunctures, ear':ab,ti OR 'ear acupunctures':ab,ti OR 'auricular acupuncture':ab,ti OR 'ear acupuncture':ab,ti OR 'acupuncture, auricular':ab,ti OR 'acupunctures, auricular':ab,ti OR 'auricular acupunctures':ab,ti OR 'acupuncture point':ab,ti OR 'point, acupuncture':ab,ti OR 'points, acupuncture':ab,ti OR 'acupoints':ab,ti OR 'acupoint':ab,ti OR 'analgesia, acupuncture':ab,ti OR 'acupuncture anesthesia':ab,ti OR 'anesthesia, acupuncture':ab,ti | 16196 |
| #9 | #7 OR #8 | 43322 |
| #10 | #3 AND #6 | 9107 |
| #11 | 'randomized controlled trial'/exp | 809052 |
| #12 | 'clinical trials, randomized':ab,ti OR 'trials, randomized clinical':ab,ti OR 'controlled clinical trials, randomized':ab,ti | 252 |
| #13 | #11 OR #12 | 809276 |
| #14 | #9 AND #10 AND #13 | 9 |
| #15 | #9 AND #10 AND #13 | 9 |

**Table 3:** Web of Science Search

| **NO** | **Search Details** | **Results** |
| --- | --- | --- |
| #1 | Acupuncture OR Acupuncture Therapy OR Acupuncture, Ear OR Acupuncture Points OR Acupuncture Analgesia OR Pharmacopuncture OR Acupuncture Treatment OR Acupuncture Treatments OR Treatment, Acupuncture OR Pharmacoacupuncture Treatment OR Pharmacoacupuncture Therapy OR Acupotomies OR Acupuncture Point OR Acupoint OR Acupuncture Anesthesia OR Anesthesia, Acupuncture (Topic) and Preprint Citation Index (Exclude – Database) | 94660 |
| #2 | (TS=(Parkinson Disease)) AND TS=(Parkinson Disease OR Secondary Parkinson Disease OR Symptomatic Parkinson Disease OR Parkinsonism, Symptomatic OR Symptomatic Parkinsonism OR Secondary Parkinsonism OR Parkinson Disease, Symptomatic OR Parkinsonism, Secondary) and Preprint Citation Index (Exclude – Database) | 323942 |
| #3 | TS=(Anxiety OR Angst OR Social Anxiety OR Anxieties, Social OR Social Anxieties OR Hypervigilance OR Nervousness OR Anxiousness) and Preprint Citation Index (Exclude – Database) | 725365 |
| #4 | #3 AND #2 and Preprint Citation Index (Exclude – Database) | 15912 |
| #5 | TS=(Randomized Controlled Trials OR Clinical Trials, Randomized OR Trials, Randomized Clinical OR Controlled Clinical Trials, Randomized) and Preprint Citation Index (Exclude – Database) | 1040283 |
| #6 | #4 AND #5 AND #1 and Preprint Citation Index (Exclude – Database) | 11 |

**Table 4:** Cochrane Library Search

| **NO** | **Search Details** | **Results** |
| --- | --- | --- |
| #1 | MeSH descriptor: [Parkinson Disease] explode all trees | 6045 |
| #2 | (Idiopathic Parkinson's Disease):ti,ab,kw OR (Lewy Body Parkinson's Disease):ti,ab,kw OR (Parkinson's Disease, Idiopathic):ti,ab,kw OR (Parkinson's Disease, Lewy Body):ti,ab,kw OR (Parkinson Disease, Idiopathic):ti,ab,kw OR (Parkinson's Disease):ti,ab,kw OR (Idiopathic Parkinson Disease):ti,ab,kw OR (Lewy Body Parkinson Disease):ti,ab,kw OR (Primary Parkinsonism):ti,ab,kw OR (Parkinsonism, Primary):ti,ab,kw OR (Paralysis Agitans):ti,ab,kw | 13091 |
| #3 | #1 OR #2 | 13091 |
| #4 | MeSH descriptor: [Anxiety] explode all trees | 12544 |
| #5 | (Angst):ti,ab,kw OR (Social Anxiety):ti,ab,kw OR (Anxieties, Social):ti,ab,kw OR (Anxiety, Social):ti,ab,kw OR (Social Anxieties):ti,ab,kw OR (Hypervigilance):ti,ab,kw OR (Nervousness):ti,ab,kw OR (Anxiousness):ti,ab,kw | 13262 |
| #6 | #4 OR #5 | 23979 |
| #7 | #3 and #6 | 143 |
| #8 | MeSH descriptor: [Acupuncture Therapy] explode all trees | 7041 |
| #9 | (Pharmacopuncture):ti,ab,kw OR(Acupuncture Treatment):ti,ab,kw OR(Acupuncture Treatments):ti,ab,kw OR(Treatment, Acupuncture):ti,ab,kw OR(Pharmacoacupuncture Treatment):ti,ab,kw OR(Therapy, Acupuncture):ti,ab,kw OR(Treatment, Pharmacoacupuncture):ti,ab,kw OR(Pharmacoacupuncture Therapy):ti,ab,kw OR(Therapy, Pharmacoacupuncture):ti,ab,kw OR(Acupotomy):ti,ab,kw OR(Acupotomies):ti,ab,kw OR(Acupunctures, Ear):ti,ab,kw OR(Ear Acupunctures):ti,ab,kw OR(Auricular Acupuncture):ti,ab,kw OR(Ear Acupuncture):ti,ab,kw OR(Acupuncture, Auricular):ti,ab,kw OR(Acupunctures, Auricular):ti,ab,kw OR(Auricular Acupunctures):ti,ab,kw OR(Acupuncture Point):ti,ab,kw OR(Point, Acupuncture):ti,ab,kw OR(Points, Acupuncture):ti,ab,kw OR(Acupoints):ti,ab,kw OR(Acupoint):ti,ab,kw OR(Analgesia, Acupuncture):ti,ab,kw OR(Acupuncture Anesthesia):ti,ab,kw OR(Anesthesia, Acupuncture):ti,ab,kw | 19792 |
| #10 | #8 OR #9 | 20162 |
| #11 | MeSH descriptor: [Randomized Controlled Trial] explode all trees | 37 |
| #12 | (Clinical Trials, Randomized):ti,ab,kw OR(Trials, Randomized Clinical):ti,ab,kw OR (Controlled Clinical Trials, Randomized):ti,ab,kw OR( Randomized):ti,ab,kw | 1163647 |
| #13 | #11 OR #12 | 1163647 |
| #14 | #7 and #10 and #13 | 2 |

**Table 5:** CNKI Search

| **NO** | **Search Details** | **Results** |
| --- | --- | --- |
| #1 | （主题：帕金森）OR（主题：帕金森病）OR（主题：帕金森）OR（主题：震颤麻痹）OR（主题：帕金森综合征）AND（主题：焦虑）OR（主题：社交焦虑）OR（主题：紧张）OR（主题：焦急）OR（主题：焦虑）OR（主题：过度警觉）AND（主题：针灸）OR（主题：针刺）OR（主题：电针）OR（主题：耳针）OR（主题：头针）OR（主题：针灸疗法）OR（主题：针刺治疗）OR（主题：穴位按压）OR（主题：穴位刺激）AND（篇关摘：随机对照(精确)）OR（篇关摘：随机(精确)）OR（篇关摘：RCT(精确)）OR（篇关摘：随机对照试验(精确) | 6 |

**Table 6:** Wanfang Search

| **NO** | **Search Details** | **Results** |
| --- | --- | --- |
| #1 | 主题:(帕金森+帕金森病+帕金森综合征+震颤麻痹) and主题:(焦虑+社交焦虑症+紧张+过度警觉) and主题:(针灸治疗+针灸+针灸疗法+针刺+针刺疗法+电针+头针+耳针+微针+穴位刺激+穴位按摩) and主题:(随机对照试验+随机+RCT+随机对照) | 0 |

**Table 7:** CBM Search

| **NO** | **Search Details** | **Results** |
| --- | --- | --- |
| #1 | "帕金森障碍"[不加权:扩展] | 12407 |
| #2 | (( "帕金森"[常用字段:智能] OR "帕金森综合征"[常用字段:智能]) OR "震颤麻痹"[常用字段:智能]) | 180427 |
| #3 | #1 OR #2 | 180427 |
| #4 | "焦虑症"[不加权:扩展] | 123513 |
| #5 | "焦虑"[常用字段:智能] OR "社交焦虑症"[常用字段:智能] OR "紧张"[常用字段:智能] OR "过度警觉"[常用字段:智能] | 632344 |
| #6 | #4 OR #5 | 695207 |
| #7 | #3 AND #6 | 4589 |
| #8 | "针灸疗法"[不加权:扩展] | 175335 |
| #9 | 针灸"[常用字段:智能] OR "针刺"[常用字段:智能] OR "电针"[常用字段:智能] OR "针刺疗法"[常用字段:智能] OR "针灸治疗"[常用字段:智能] OR "头针"[常用字段:智能] OR "耳针"[常用字段:智能] OR "穴位刺激"[常用字段:智能] OR "微针"[常用字段:智能] 322069 2024-02-12 00:07:07.0 | 322069 |
| #10 | #7 OR #8 | 322069 |
| #11 | "随机对照试验"[不加权:扩展] | 202009 |
| #12 | "随机对照"[常用字段:智能] OR "随机"[常用字段:智能] OR "RCT"[常用字段:智能] | 2252649 |
| #13 | #10 OR #11 | 2252866 |
| #14 | #7 AND #10 AND #13 | 24 |

**Tble8：**维普网

| **NO** | **Search Details** | **Results** |
| --- | --- | --- |
| #1 | ((((((题名或关键词=帕金森 OR 题名或关键词=帕金森病) OR 题名或关键词=震颤麻痹) OR 题名或关键词=帕金森综合征) AND (((((题名或关键词=焦虑 OR 题名或关键词=社交焦虑) OR 题名或关键词=紧张) OR 题名或关键词=焦急) OR 题名或关键词=焦虑) OR 题名或关键词=过度警觉)) AND ((((((((题名或关键词=针灸 OR 题名或关键词=针刺) OR 题名或关键词=电针) OR 题名或关键词=耳针) OR 题名或关键词=头针) OR 题名或关键词=针灸疗法) OR 题名或关键词=针刺治疗) OR 题名或关键词=穴位按压) OR 题名或关键词=穴位刺激)) AND (((文摘=随机对照 OR 文摘=随机) OR 文摘=RCT) OR 文摘=随机对照试验)) | 2 |
