# Supplementary figures and images for: Efficacy of acupuncture in ameliorating anxiety in Parkinson's disease: a systematic review and meta-analysis with trial sequential analysis
Source: Front Aging Neurosci. 2024 Nov 11;16:1462851. doi: 10.3389/fnagi.2024.1462851 (PMC11586373; doi:10.3389/fnagi.2024.1462851)

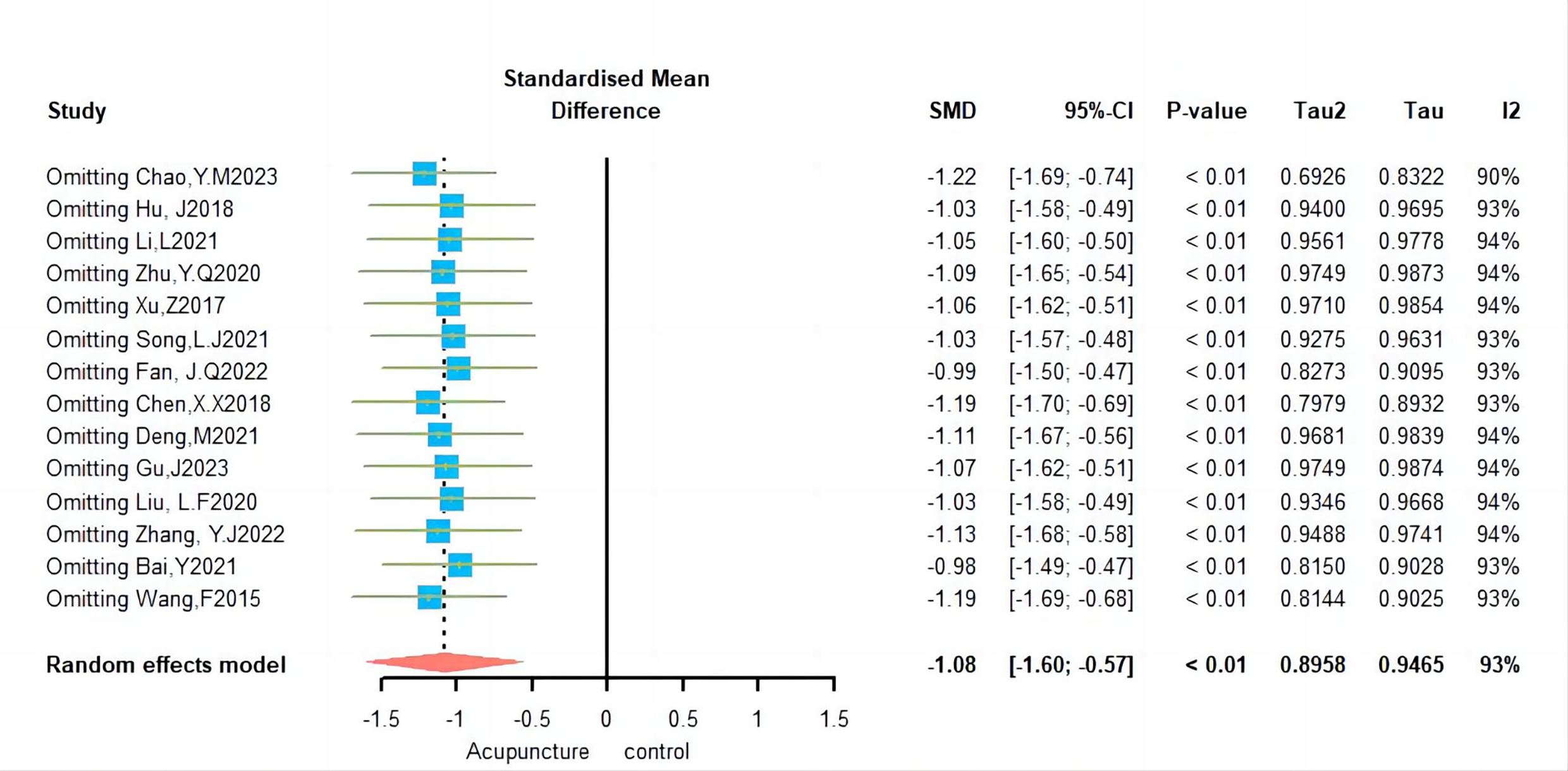

Supplement: Supplementary file 2 [file Image_1.tif]

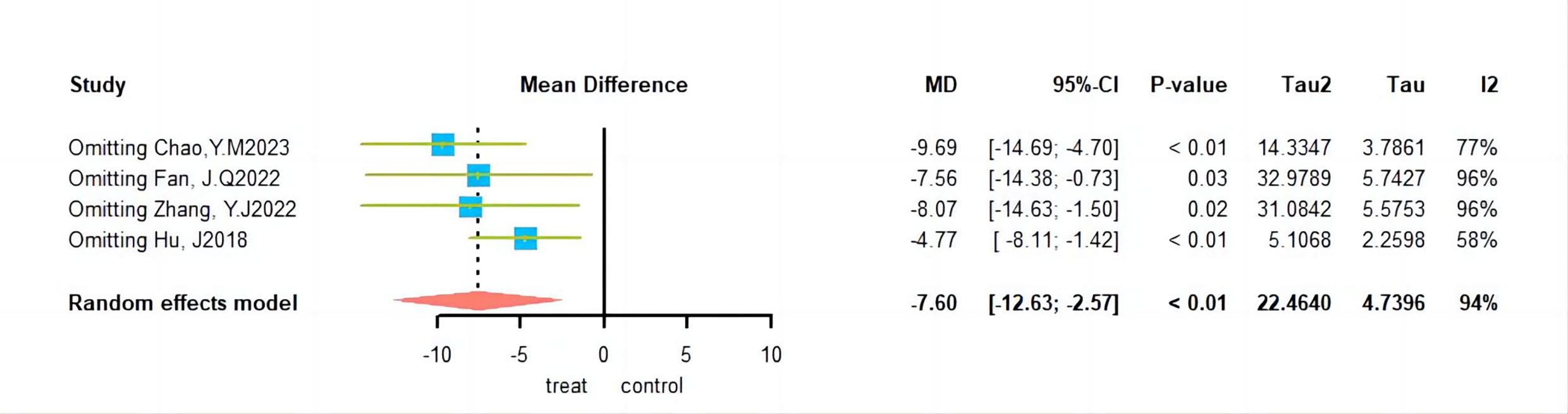

Supplement: Supplementary file 3 [file Image_2.tif]

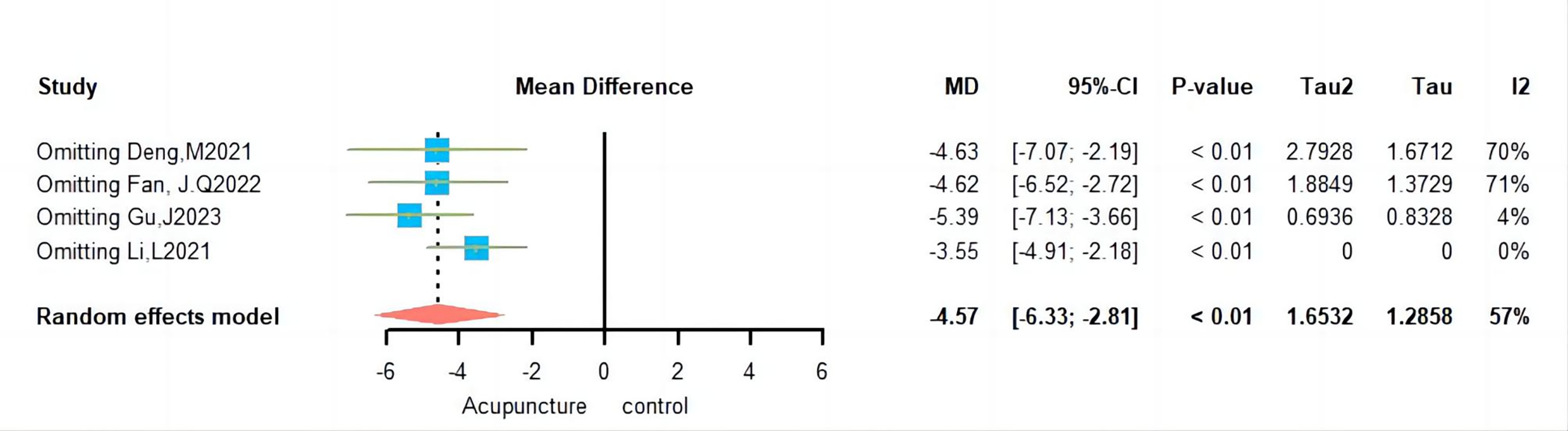

Supplement: Supplementary file 4 [file Image_3.tif]

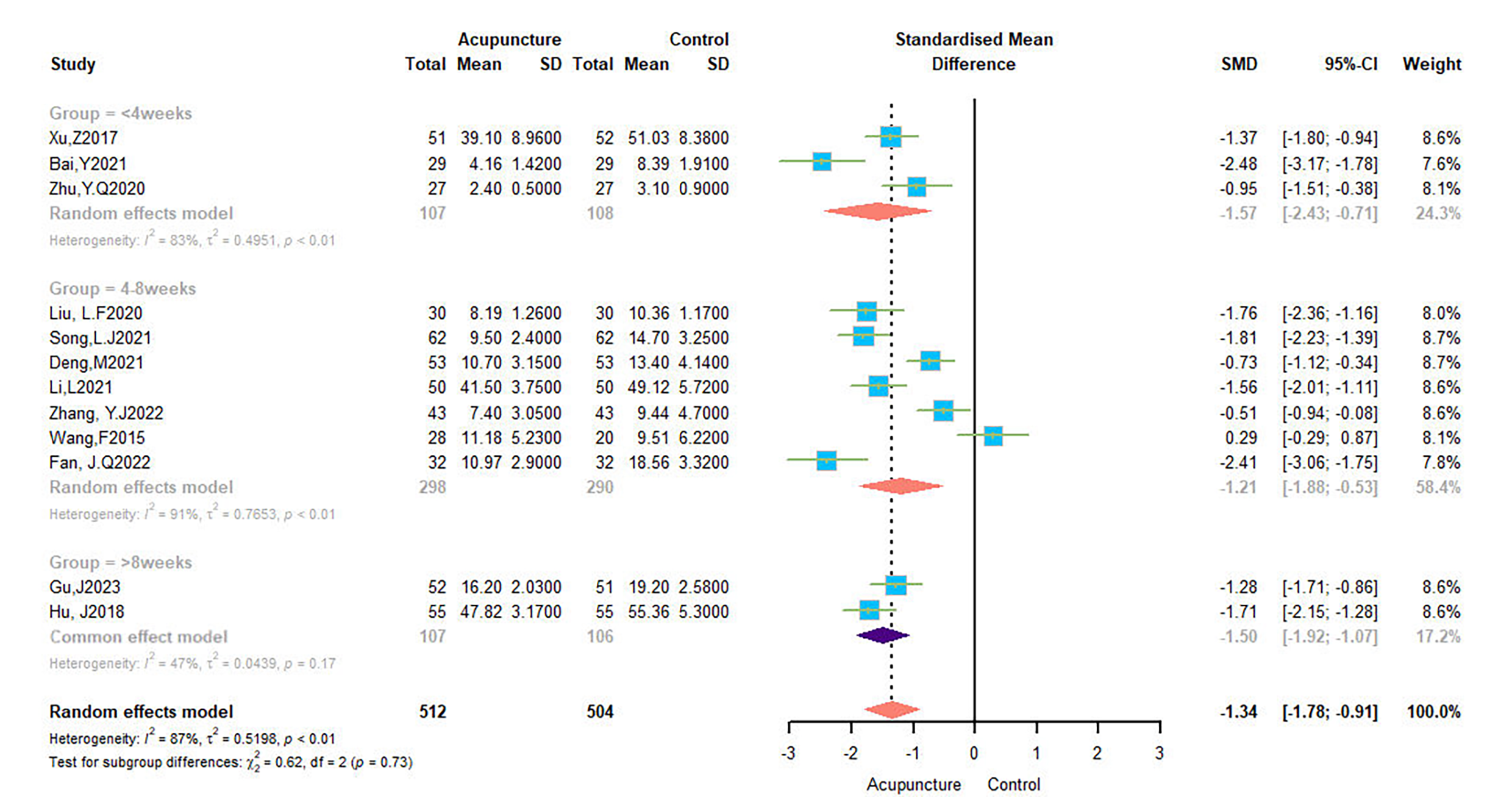

Supplement: Supplementary file 5 [file Image_4.tif]

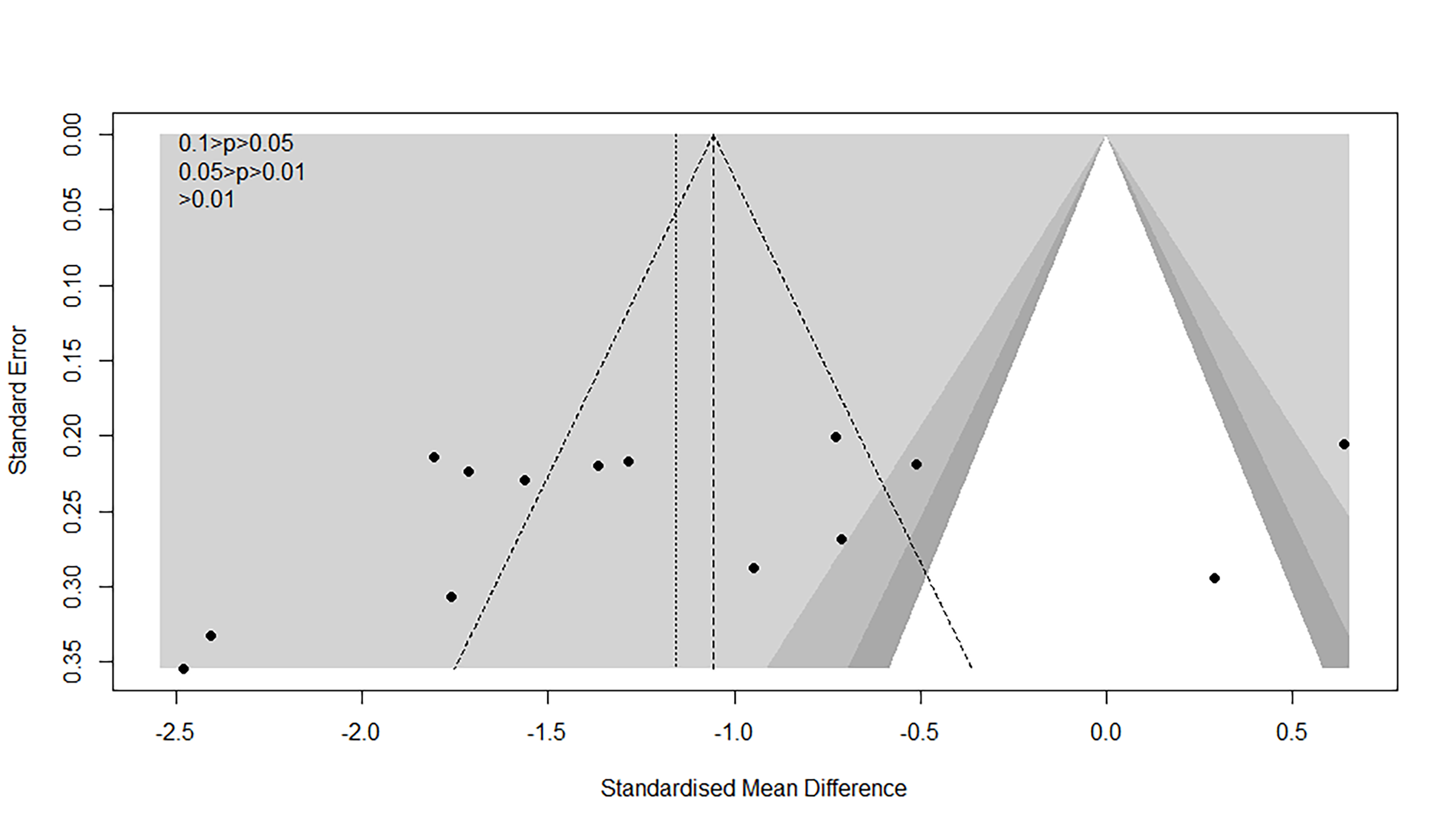

Supplement: Supplementary file 6 [file Image_5.tif]
